# An interpretable deep learning framework identifies proteomic drivers of Alzheimer’s disease

**DOI:** 10.3389/fcell.2024.1379984

**Published:** 2024-09-17

**Authors:** Elena Panizza, Richard A. Cerione

**Affiliations:** ^1^ Department of Molecular Medicine, Cornell University, Ithaca, NY, United States; ^2^ Department of Chemistry and Chemical Biology, Cornell University, Ithaca, NY, United States

**Keywords:** Alzheimer’s disease, machine learning, proteomics, clinical data, aging, disease drivers

## Abstract

Alzheimer’s disease (AD) is the leading neurodegenerative pathology in aged individuals, but many questions remain on its pathogenesis, and a cure is still not available. Recent research efforts have generated measurements of multiple omics in individuals that were healthy or diagnosed with AD. Although machine learning approaches are well-suited to handle the complexity of omics data, the models typically lack interpretability. Additionally, while the genetic landscape of AD is somewhat more established, the proteomic landscape of the diseased brain is less well-understood. Here, we establish a deep learning method that takes advantage of an ensemble of autoencoders (AEs) — EnsembleOmicsAE–to reduce the complexity of proteomics data into a reduced space containing a small number of latent features. We combine brain proteomic data from 559 individuals across three AD cohorts and demonstrate that the ensemble autoencoder models generate stable latent features which are well-suited for downstream biological interpretation. We present an algorithm to calculate feature importance scores based on the iterative scrambling of individual input features (i.e., proteins) and show that the algorithm identifies signaling modules (AE signaling modules) that are significantly enriched in protein–protein interactions. The molecular drivers of AD identified within the AE signaling modules derived with EnsembleOmicsAE were missed by linear methods, including integrin signaling and cell adhesion. Finally, we characterize the relationship between the AE signaling modules and the age of death of the patients and identify a differential regulation of vimentin and MAPK signaling in younger compared with older AD patients.

## 1 Introduction

Alzheimer’s disease (AD) is a degenerative disease of the brain that presents with decline of multiple cognitive areas including memory, language, spatial recognition, sociality, behavior, and motor functions (Scheltens et al., 2021). The majority of AD cases occur in individuals over the age of 65 that do not carry predisposing mutations (sporadic, late-onset AD, about 99% of the reported cases) (AD facts and figures, 2024). As life expectancy grows across the globe, increasing numbers of individuals are developing AD (Hou et al., 2019) and a cure is direly needed. Several factors are known to contribute to the progression of AD, including alterations of the brain cells (De Strooper and Karran, 2016) and vasculature (Sweeney et al., 2018), inflammation (Sweeney et al., 2018), accumulation of amyloid beta and hyperphosphorylated tau (Long and Holtzman, 2019), and genetic predisposition (Gatz et al., 2006; Jansen et al., 2019). However, the causes underlying the disease are still not known (Breijyeh et al., 2020).

In recent years, comprehensive genomics, epigenomics, transcriptomics, proteomics (Higginbotham et al., 2020), and metabolomics profiles of *postmortem* brain and liquid biopsy samples have been obtained from large cohorts of AD and control individuals (Allen et al., 2016; Bai et al., 2020; Bennett et al., 2018; Wang M et al., 2018). The analysis of large-scale molecular data can provide important insights on drivers of AD. Machine learning methods are particularly well-suited for the analysis of complex data and have been shown to be able to identify distinct signaling nodes compared with linear methods (Beebe-Wang et al., 2021). Important advances have been made in the field of analyzing brain images with the aid of deep learning (Jo et al., 2019), including the recent development of a model that can distinguish abnormal brain connections occurring at earlier or later stages of AD from magnetic resonance images (Zuo et al., 2021). More recently, the same authors presented a unified framework to integrate structural and functional brain images to predict the stage of AD with high accuracy (Zuo et al., 2023). In terms of the applications of deep learning to the analysis of molecular expression data, the models typically suffer from poor interpretability, which makes it difficult to understand the underlying biology (Ching et al., 2018; Panizz, 2023). Additionally, previous studies focused primarily on the analysis of RNA-sequencing data (Beebe-Wang et al., 2021), while an effort to integrate and interpret proteomics data from *postmortem* brain samples across multiple patient cohorts is, to date, lacking.

Since protein levels can be regulated independently from their transcripts (Aebersold et al., 2018; Li and Vacanti, 2023), protein abundances offer a functional snapshot of cellular states. Here, we focused on the study of the brain proteome to gain insights into the cellular functions that are affected in AD. We present EnsembleOmicsAE, a deep learning-based framework to integrate multiple AD datasets and identify the biological nodes that drive the disease phenotype. Our model relies on a type of neural network called an autoencoder, which is well-suited to identify important signaling nodes as it learns a condensed representation of the data (Simidjievski et al., 2019). Autoencoders include several layers each containing multiple computational units or “neurons.” The layers are arranged to form an “hourglass” architecture, which includes two identical mirror-imaged components called the encoder and the decoder, connected by a small central layer termed the latent layer (Y. Wang et al., 2016). In the latent layer, the model learns a set of “latent features,” which are a condensed representation of the data, holding the key information for reconstructing the input in the decoder. Studies on neural networks in the field of image recognition have clarified that neurons within shallower layers are able to detect simple features, while neurons within deeper layers learn to detect progressively more complex features (Lee et al., 2009). Therefore, we reasoned that each neuron within the latent layer should learn to detect high-level biological features that are important for describing the difference between the control and the AD brain proteomes. For this reason, we set out to develop an algorithm to quantify the contributions of individual proteins relative to each latent feature learned by the neurons in the latent layers. This aspect of our algorithm is innovative because it identifies features that are important for individual neurons, as opposed to existing methods, which identify features that are important for the model as a whole (Ching et al., 2018). Finally, our analysis identifies a signaling hub that is over-activated in younger compared with older AD patients and involves a regulation of the cellular differentiation process operated by the protein vimentin (VIM) and the mitogen-activated protein kinases MAPK1 and MAPK3.

## 2 Materials and methods

### 2.1 Bioinformatic analyses, statistics, and data visualization

All analyses were conducted using Python (Rossum and Drake, 2009). Plots were generated using the libraries Seaborn (Waskom, 2021) and Matplotlib (Hunter, 2007). GO enrichment analysis was performed using the package GOATOOLs (Klopfenstein et al., 2018), and Fisher’s exact test was employed to evaluate the significance of the enrichment. The web-based Search Tool for the Retrieval of Interacting Genes/Proteins (STRING; https://string-db.org) (Szklarczyk et al., 2017) was employed for protein–protein interaction analysis. Details on applied statistical analyses are specified in the figures and corresponding legends.

### 2.2 Data processing

To develop the EnsembleOmicsAE model, we used the following proteomics datasets available through the AD Knowledge Portal (https://adknowledgeportal.synapse.org/Explore/Data). The first dataset is the tandem mass tag (TMT) proteomics data (syn22277191) from the Banner Sun Health Research Institute (Banner) study. The second dataset is the TMT proteomics data (syn26051803) from the Religious Orders Study and Memory and Aging Project (ROSMAP) study. The third dataset is the TMT proteomics data (syn24983526) from the Mount Sinai Brain Bank (MSBB) study. To pre-process the data, we performed the following steps. First, protein abundances represented as TMT ratios were log_2_-transformed, if they were not already. Proteins that had more than 20% missing expression values were removed. In the cases where Ensemble gene IDs or UniProt IDs were provided, the identifiers were converted to gene symbols. When multiple proteins corresponded to the same gene name, we collapsed them to a single gene-centric feature by taking their median expression value representing more robust quantifications, as well as to facilitate downstream biological interpretation. The lists of all genes mapping to multiple identifiers are provided in Supplementary Tables S1–S3. Protein abundances were normalized to the median expression value of all proteins for each patient, to assume an equal amount of total protein analyzed for each sample. Protein abundances were further normalized relative to the median expression value of all patients for each protein, to adjust for differences in baseline protein abundance and facilitate the identification of group of proteins that follow similar patterns of regulation. Outlier samples were identified using the Mahalanobis distance, and samples with a distance greater than one standard deviation over the mean distance were removed. After data pre-processing, the datasets were combined and the features that were common across the three datasets were kept (Supplementary Figure S1A). This yielded a dataset containing 673 samples and 6,362 features, including 6,360 quantified proteins and two clinical features, age at death, and sex. The labels were the diagnosis of the patients, which was either control, mild cognitive decline (MCI), or AD. The three datasets contributed respectively: Banner: control, 89; AD, 86; ROSMAP Round 1: control, 151; AD, 101; MCI, 91; MSBB: AD, 106; control, 26; MCI, 23. After combining all datasets, the protein abundances were standardized to have similar distribution in the three sets, by subtracting the median value from each individual feature value and dividing by the standard deviation of the values for the same feature. Then, all values were normalized to the median value for each sample, to assume equal sample loading. The median values were used for the standardization and normalization steps, to obtain robust estimates of protein abundance that are less influenced by outliers. Finally, each clinical feature was transformed to numerical values with a distribution similar to that of the protein expression data. Specifically, z-score normalization was applied to the values for patients’ age at death. Sex was encoded as the negative inter-quartile range value for male subjects and the positive inter-quartile range value for female subjects (Supplementary Figure S1E). UMAP analysis highlighted a poor separation between the three sample groups (silhouette score of the UMAP embedding was 0.01, Supplementary Figure S1B), while removal of the MCI samples yielded a UMAP silhouette score of 0.15 and a visually clear separation of the control and AD samples (Supplementary Figure S1C). The MCI samples were therefore removed from subsequent analysis, and the final combined dataset contained 559 samples described by 6,362 features, including 6,360 quantified proteins and 2 clinical features, namely, age at death and sex.

### 2.3 Feature selection

**FIGURE 1:**
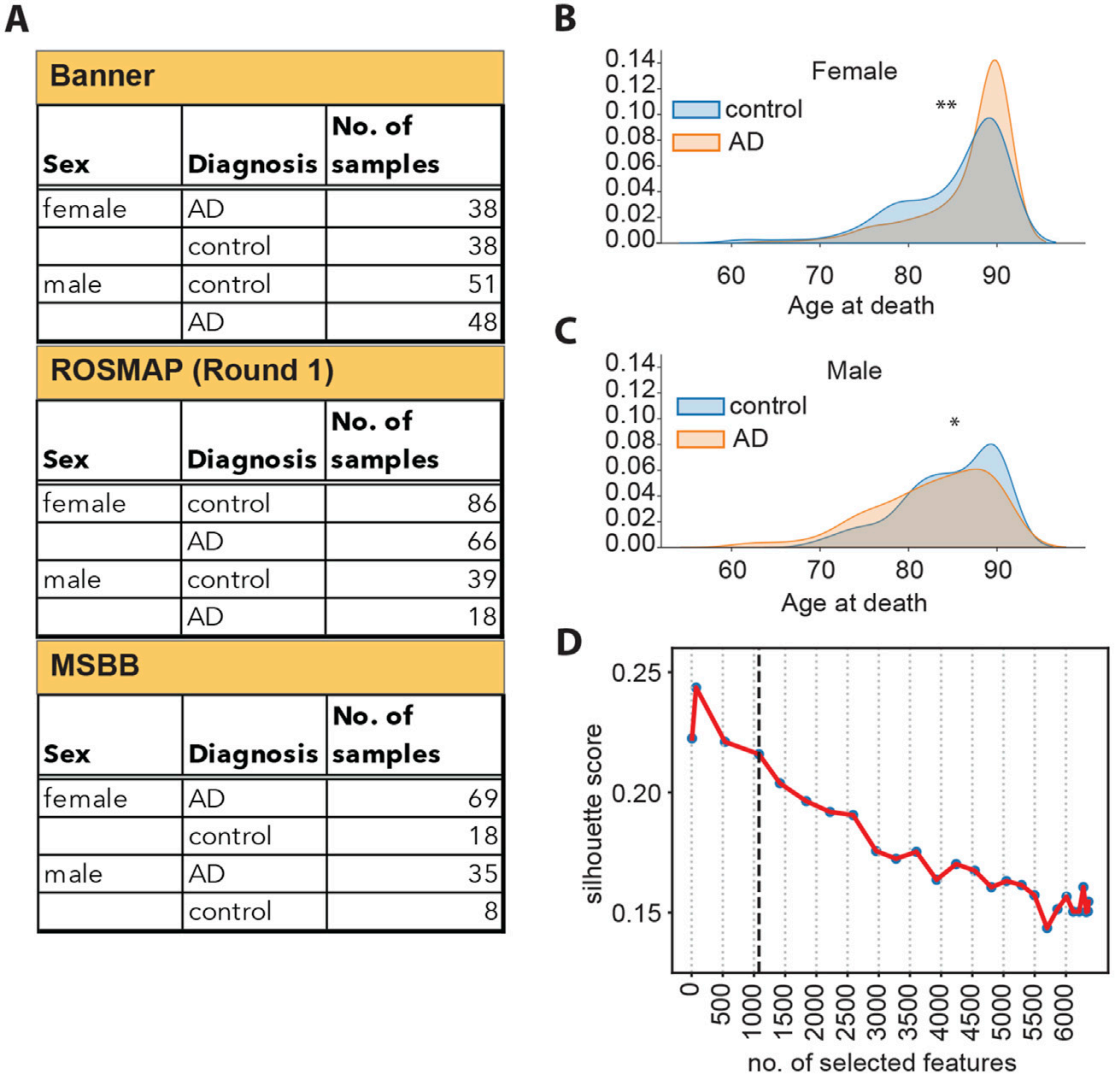
**(A)** Schematic overview of samples available from each cohort, split by sex and diagnosis. **(B)** Density plots representing the distribution of the age at death for female donors, broken down by diagnosis. **: Student’s t-test *p*-value <5*10^−3^. **(C)** Density plots representing the distribution of the age at death for male donors, broken down by diagnosis. *: Student’s t-test *p*-value <1*10^−2^. **(D)** Silhouette scores of the UMAP embedding for datasets of different sizes, as indicated on the *x*-axis. The vertical dashed line corresponds to 1,080 features.

Feature selection was performed independently on the female and male datasets to preserve sex-specific molecular events linked with AD. Additionally, we applied two approaches, i.e., the f-statistic and the mutual information algorithm, to select features that are associated with the outcome (control or AD) both in a linear and non-linear manner. After separating the combined dataset into a female and a male set, we applied the ANOVA F-test to rank the proteins based on the extent to which their expression levels are statistically different between the control and diseased groups. Additionally, the mutual information (MI) score (Kraskov et al., 2004) between each individual features and the label (control or AD) was used to rank the features in the female and male datasets. To test what would be an optimal number of features to select, we created sets of selected features of different sizes ranging from 7 to 6,360. For each set, 5% of the total number of features selected comprised the top-ranking features using the MI score in the female dataset, and another 5% comprised the top-ranking features using the MI score in the male dataset; the rest of the features were selected using the ANOVA F-test statistic. For each of the selected features of different size, we calculated 1) the cumulative variance explained by the first two principal components based on PCA and 2) the UMAP silhouette score describing the separation between the control and the AD groups described by the set being analyzed (Figure 1D; Supplementary Figure S2D). The set of 1,080 features selected for subsequent analyses is the union of the top 585 proteins selected with the ANOVA F-test for the male and female datasets and the top 54 (5% of 1,080) features ranked by their MI score in the male and female datasets separately.

### 2.4 Training and evaluation of the autoencoder models

**FIGURE 2:**
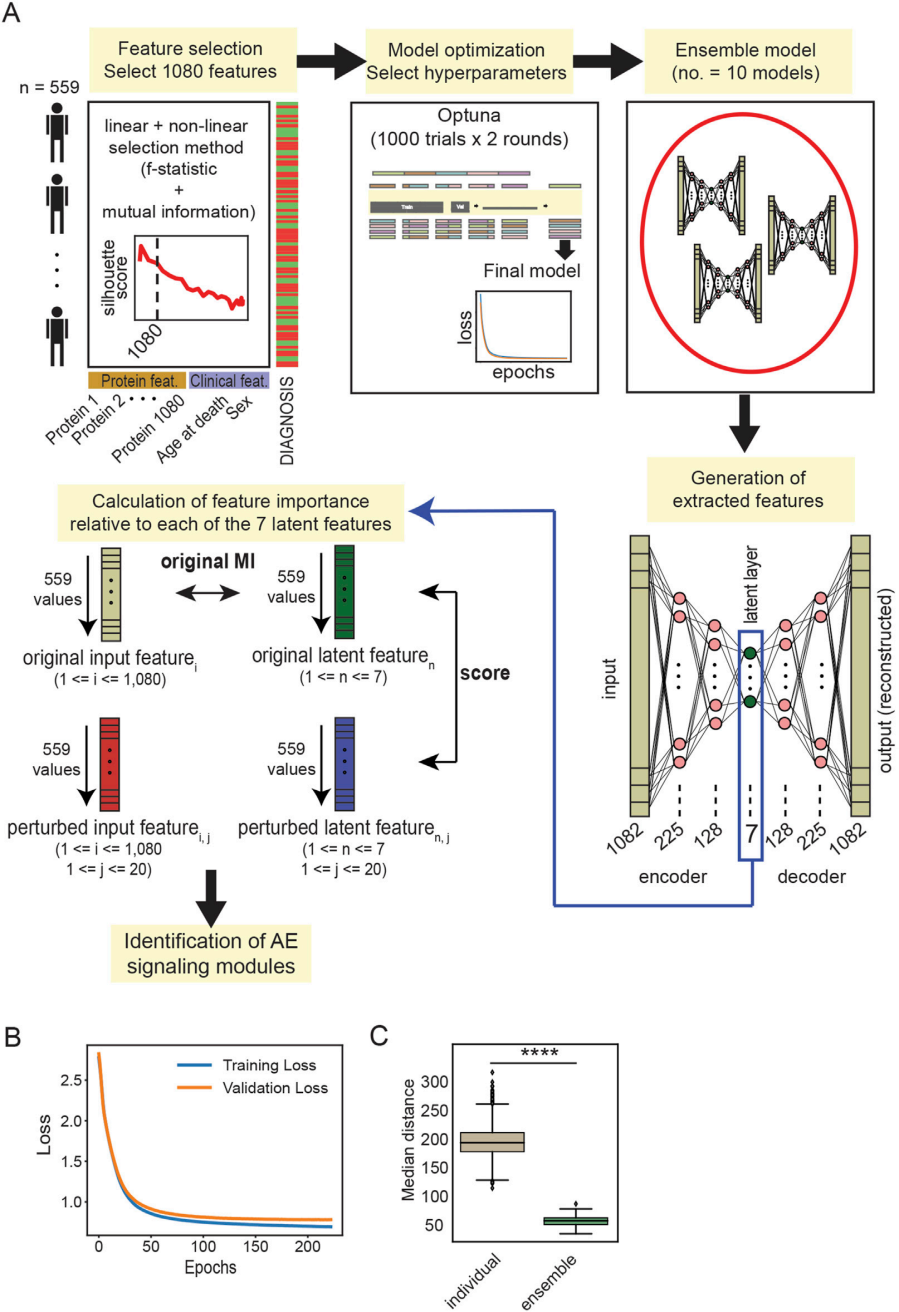
**(A)** Schematic representation of the workflow employed for EnsembleOmicsAE. For the calculation of the feature importance scores, the number of input protein features is indicated with *i* (ranging between 1 and 1,080); the number of latent features is indicated with *n* (ranging between 1 and 7); and the number of iterations to generate the perturbed input features is indicated with *j* (ranging between 1 and 20) (see Methods). **(B)** Training and validation loss, measured as the reconstruction error, of a representative autoencoder trained using the set of hyperparameters selected after the two optimization rounds. **(C)** Box plot representing the median Euclidian distance between every pair of latent features extracted using individual models and ensemble models. ****: Student’s t-test *p*-value <1*10^−99^.

All autoencoder models were built using the TensorFlow (Abadi et al., 2016) and Keras packages. We constructed our network to include an encoder and a decoder, which are identical mirror copies of each other. The decoder contains the input layer, two hidden layers, and a latent layer. The latent layer is both the last layer for the encoder and the first layer of the decoder. The decoder then mirrors the encoder, i.e., it contains two hidden layers and an output layer which have the same number of neurons as those in the encoder (Figure 2A). The neurons in the network layers are loaded with rectified linear unit (ReLU) activation functions (Agarap, 2018). Each model was trained for a maximum of 300 epochs with early stopping set to a value for patience of 10 and the option to restore the best weights. The batch size was set to 16, and we applied the Adam optimizer (Kingma and Ba, 2014), with the option to clip gradients at the value of 0.1. These settings were selected because they provided a good level of stability of the models, allowing for the extensive hyperparameter tuning process described in the Results section (Figure 2A). For all models, the reconstruction loss is measured as the mean squared error of the reconstructed data generated by the output layer compared to the original data that were used as the input. The UMAP embedding of the latent layer extracted from training on the whole training set is recorded for each set of hyperparameters that are tested. The ensemble models are constructed by combining 10 individual encoder models. To generate the ensemble latent features, the latent features generated with each individual encoder model are averaged with the keras.layers.Average () function. This function computes the element-wise average of the latent representation and produces a single averaged tensor which contains the ensemble latent features.

### 2.5 Feature importance scores and generation of AE signaling modules

To calculate the importance of the input features (i.e., the proteins) relative to each neuron in the latent layer, we devised the following algorithm:

Perturbed data and score calculation:

$$for\;n\in \left\{0,\ldots ,N-1\right\},for\;i\in \left\{0,\ldots ,I-1\right\},for\;j\in \left\{0,\ldots ,J-1\right\}:$$

$$perturbed\_latent_{i,n,j}=\text{ensemble}\_\text{encoder}.\text{predict}\left(\text{shuffle}\left(datavalues:{,}_{i}\right)\right),$$ (1)

$${\text{scores}}_{i,n,j}=\text{mutual}\_\text{info}\_\text{regression}\left(original\_latent:{,}_{n},perturbed\_latent_{i,n,j}\right).$$ (2)

Original mutual information, median score, and score variability calculation:

$$for\;n\in \left\{0,\ldots ,N-1\right\},for\;i\in \left\{0,\ldots ,I-1\right\}:$$

$$original\_MI_{i,n}=\text{mutual}\_\text{info}\_\text{regression}\left(data\_values:{,}_{i},original\_latent:{,}_{n}\right),$$ (3)

$$median\_score_{i,n}=median\left(\left\{scores_{i,n,j}|j\in \{0,\ldots ,J-1\right.\}\}\right),$$ (4)

$$score\_variability_{i,n}=IQR\left(scores_{i,n,:}\right).$$ (5)

Feature selection criteria:

$$\begin{array}{l} selected\_features=\left\{i|score\_variability_{i,n}>0,median\_score_{i,n}\right.\\ \leq \text{quantile}\left(median\_score,0.3\right),original\_MI_{i,n}\\ \left.\geq \text{quantile}\left(original\_MI,0.6\right)\right\}. \end{array}$$ (6)

ensemble_encoder: the encoder model generated by combining 10 individual encoder models.

*data_values*: matrix of input data where each column represents an input feature (i.e., a protein) and each row represents a sample.

*original_latent*: matrix of latent features generated by the encoder part of the autoencoder. Each column represents a latent feature, and each row represents a sample.

i: index for input features in *data_values*. It ranges from 0 to number of input features—1.

n: index for latent features in *original_latent*. It ranges from 0 to number of latent features—1.

j: index for the number of perturbation iterations (1≤j ≤ 20).

I, N, and J: ranges representing the total number of input features (I = 1,080), latent features (N = 7), and iterations (J = 20), respectively. The shuffle () function is applied to the *i*th column in each iteration (j) for each latent feature (n).

*perturbed_latent*: 3D array of perturbed latent features, with the same structure as *original_latent,* plus a third dimension corresponding to the iterations of perturbation (j).

*scores*: 3D array containing the mutual information scores, where the third dimension corresponds to iterations of perturbation (j).

mutual_info_regression (): function from the library scikit-learn (Pedregosa et al., 2011) which estimates mutual information (MI) (Kraskov et al., 2004) between two continuous variables. MI is a non-negative value which measures the degree of dependency between the variables. It equals to zero when the two variables are independent, while the higher the value, the higher the dependency.

shuffle (): function from the library NumPy (numpy.random.shuffle) (Harris et al., 2020), which shuffles the content of an array.

median (): function from the library NumPy (numpy.median) (Harris et al., 2020), which computes the median of the provided data.

To calculate the importance of each protein in determining the condensed data representation (i.e., the latent features), we applied the following approach for each of the proteins that are used as an input for the model. The expression values for one protein were randomly scrambled across the 559 samples, in order to alter the relation between protein expression levels and diagnosis. The scrambled values were re-positioned within the set of all proteins (1,080 protein expression values, across the 559 samples). Then, the ensemble encoder was used to predict new latent features (*perturbed_latent*, Equation 1). A score was calculated as the MI value between the original and the perturbed latent features; the lower the score, the greatest the impact of shuffling a particular feature (Equation 2). The original MI value was calculated between the expression values for the protein being examined (not shuffled) and the original latent features (the higher the score, the more important the input feature, Equation 3). The shuffling operation was iterated 20 times for each protein, and the median score was calculated as the median of the scores calculated at each iteration (Equation 4). For each protein, the score variability was calculated as the inter-quartile range of the score values across the 20 iterations of shuffling (Equation 5). Finally, proteins were selected as important if their median score is below or equal to the 30th percentile of all median score values; their original MI value is above or equal to the 60th percentile of all original MI values; and their score variability is greater than zero (Equation 6; Supplementary Figure S5B). The proteins that were selected as important within each latent features were included in the respective AE signaling modules. The ≈100 top-ranking proteins for each AE signaling module, which were used for the protein–protein interaction analysis, were obtained by applying the following conservative thresholds: the median score is below or equal to the 20th percentile of all median score values; their original MI value is above or equal to the 80th percentile of all original MI values; and their score variability is greater than zero.

### 2.6 Linear measures of similarity and distance

**FIGURE 3:**
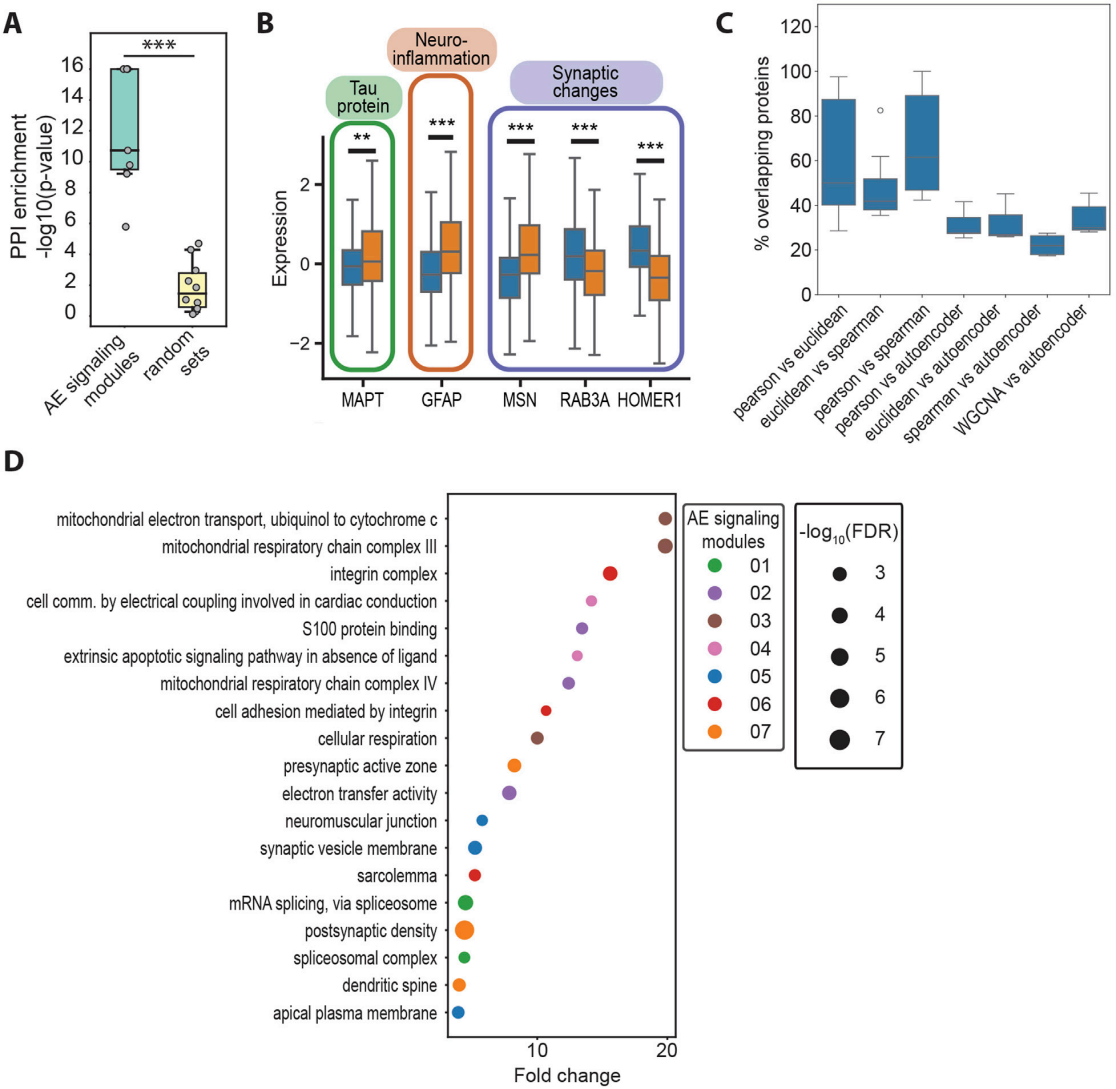
**(A)** Negative logarithm of the protein–protein interaction (PPI) enrichment values for the top genes for the AE signaling modules identified with EnsembleOmicsAE (78 < n < 102), compared with size-matched sets of genes selected at random (n = 80). ***: Student’s t-test *p*-value <1*10^−4^. **(B)** Box plots representing the protein expression levels of AD driver proteins in healthy (blue) or AD (orange) samples. **: Student’s t-test *p*-value <5*10^−3^. ***: Student’s t-test *p*-value <1*10^−4^. MAPT, microtubule-associated protein tau; GFAP, glial fibrillary acidic protein; MSN, moesin; RAB3A, Ras-related protein Rab-3A; HOMER1, Homer protein homolog 1. **(C)** Percent of overlapping proteins between the AE signaling modules and the clusters obtained with linear methods. All pair-wise overlaps between clusters and/or AE signaling modules were calculated, as detailed on the *x*-axis. The top seven overlap values (sorted in descending order) for each comparison are plotted. **(D)** GO terms that are enriched in no more than two of the AE signaling modules. For each module, terms are ranked based on their fold enrichment and *p*-value, and up to the top-three are listed. The size of the bubble is proportional to the negative logarithm of the false discovery rate of the enrichment, and the color represents the AE signaling module, in which each GO term is enriched.

To obtain the protein clusters based on linear methods (Figure 3B), the dataset containing the 1,080 selected protein features and 559 samples was clustered using Pearson correlation, Spearman correlation, and Euclidian distance with Ward linkage. The dendrograms were cut into seven clusters (to match the number of AE signaling modules) using the ‘fcluster’ and ‘maxclust’ methods from the SciPy package. Then, up to the top-189 proteins (matching the average size of the AE signaling modules) from each cluster were selected based on their correlation or distance. The proteins within each AE signaling module (Supplementary Figure S6) were clustered using Euclidian distance with Ward linkage. The dendrogram was separated into four clusters using the ‘fcluster’ and ‘maxclust’ methods from the SciPy package, and the resulting cluster maps were visualized. The dataset containing the 1,080 selected protein features and 559 samples was also clustered using weighted correlation network analysis (WGCNA) (Langfelder and Horvath, 2008). WGCNA, similar to the Multiscale Embedded Gene Co-expression Network Analysis (MEGENA) (W. M. Song and Zhang, 2015), employs a network approach to identify hubs driving protein signaling. The value for the power used to determine adjacency was selected using the pickSoftThreshold function and a range between 1 and 10 for the powerVector. After constructing a topological overlap matrix, hierarchical clustering with method ‘average’ was applied to identify modules. Finally, the strength of the correlation with the modules’ Eigengenes was used to rank genes within each module, and up to the top-189 proteins from each module were selected for comparison with the AE signaling modules.

### 2.7 RNA-seq data

RNA-sequencing data for VIM expression were obtained from the Mayo clinic and ROSMAP studies. RNA-seq data from the Mayo study represented both the cerebellum (syn5201007) and the temporal cortex (syn4650265), while data from the ROSMAP study were for the bulk brain (syn3505720). For each data set, relative RNA abundance was calculated by normalizing individual FPKM values to the median value for each protein, before log_2_ transforming them. The three datasets were then combined, and the RNA abundances were normalized to the median of the combined data for each protein. Relative RNA abundances were then plotted over patients’ age (Supplementary Figure S11A).

### 2.8 External validation: cross-referencing with healthy aging data

**FIGURE 4:**
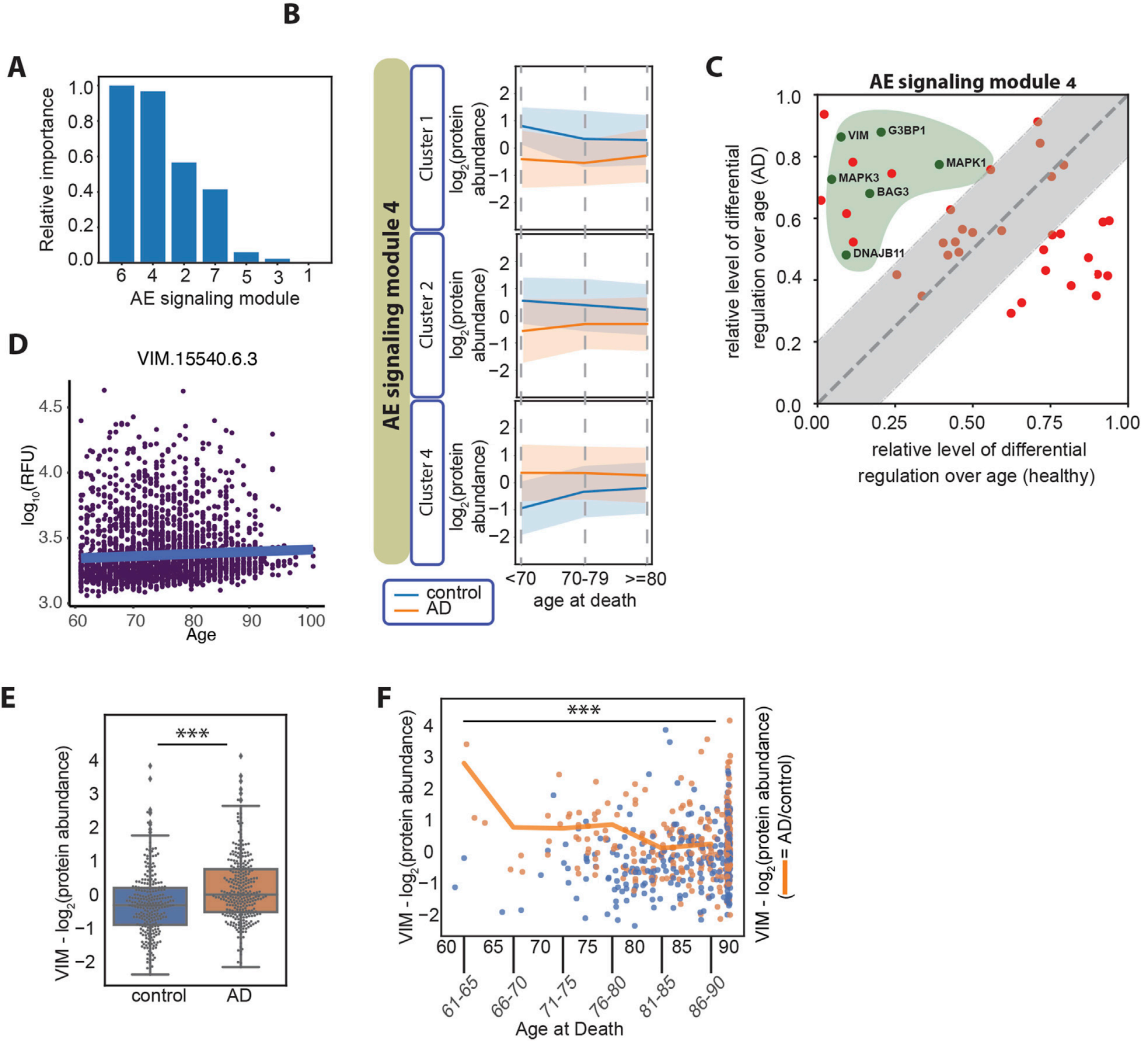
**(A)** Relative importance of age at death in relation to each of the AE signaling modules, measured as the normalized average difference between the log_2_ (AD/control) in the older and in the younger age groups (≥80 years old compared with <70 years old). The patient number per age group is age at death <70, n = 10 patients; age at death <80, n = 84 patients; age at death ≥80, n = 465. **(B)** Line plots of the three clusters in the AE signaling module no. 4, displaying differential regulation of protein expression levels in AD over patients’ age. The y-axes represent the log_2_ (protein abundance). **(C)** Differential regulation of the protein expression level over age in AD and healthy aging. Proteins overlapping between the AE signaling module no. 4 and the proteins identified by Oh et al., are represented (Oh et al., 2023) (57 proteins). The *x*-axis represents the normalized difference in protein expression levels between the older and the younger individuals in the healthy aging dataset. The y-axis represents the normalized difference in protein expression levels (AD/control) between the older and the younger age groups in the AD datasets. The gray area represents the proteins that change over age in AD and healthy aging in a similar manner (−/+ 0.2 diagonal line). The proteins marked with green dots within the green area belong to the “Bergmann glial cell differentiation” process. **(D)** Vimentin plasma protein expression levels over age in 3,800 healthy individuals (data from https://twc-stanford.shinyapps.io/aging_plasma_proteome_v2/), (Oh et al., 2023). The change in expression levels over age is 0.007, and the adjusted significance of the change is 0.08 (not significant). **(E)** Vimentin (VIM) protein expression levels in control and AD brain samples. ***: Student’s t-test *p*-value <1*10^−4^. **(F)** Vimentin protein expression levels over age. The scatter plot represents the expression levels in control samples (in blue) and AD samples (in orange), over age as a continuous variable on the *x*-axis. The line plot represents the average protein expression levels (AD/control) over the age groups as marked on the *x*-axis. The plot represents data on 559 patients in the combined set from the ROSMAP, Banner, and MSBB cohorts, including six patients between 61 and 65 years of age; five patients between 66 and 70 years of age; 33 patients between 71 and 75 years of age; 71 patients between 76 and 80 years of age; 110 patients between 81 and 85 years of age; and 334 patients 86 years and older. ***: Student’s t-test *p*-value <1*10^−4^.

Plasma protein abundances over healthy aging were obtained from Oh et al. (2023) and are available at the public site: https://twc-stanford.shinyapps.io/aging_plasma_proteome_v2/. The data represent the changes over age of 5,000 proteins measured in plasma across multiple patient cohorts. For each protein, ‘Age_beta’ represents the change in the expression level over age, and ‘Age-qval’ represents the significance of the change. The dataset was filtered to keep only the proteins that overlap with those in the set of 1,080 proteins that are used as input for the autoencoder (401 overlapping proteins). For each protein, an aging score was calculated as the sum of their rank based on ‘Age_beta’ (descending) and their rank based on ‘Age_qval’ (ascending). In practice, the aging score ranks the proteins based on having a larger and more significant change over age. A normalized aging score was then calculated by transforming all aging score values to be between 0 and 1, where values that are ranked higher are closer to 1 and values that are ranked lower are closer to 0. The normalized aging score is plotted on the *x*-axes of Figure 4C; Supplementary Figure S10B.

## 3 Results

### 3.1 EnsembleOmicsAE provides a framework to summarize omics data from multiple cohorts

The EnsembleOmicsAE model takes as input brain protein abundances and patients’ clinical data to generate a condensed representation of the data to be used for biological interpretation. The model is trained with data from the Banner Sun Health Research Institute (Banner) Study (Bai et al., 2020), the Religious Orders Study/Memory and Aging Project (ROSMAP) (Bennett et al., 2018), and the Mount Sinai Brain Bank (MSBB) study (Wang M et al., 2018), including 6,360 protein expression values over 514 distinct individuals (Figure 1A; Supplementary Figure S1A). The individuals were either healthy (control samples) or had been diagnosed with AD, while data from patients with mild cognitive decline (MCI) were excluded because they contribute negatively to sample separation (Supplementary Figures S1B and S1C). The protein expression values were normalized for study batch before combining the data and performing scaling and normalization (as detailed in the Methods section). Data for the two shared clinical features (i.e., age at death and sex) were transformed into numerical values with a distribution similar to that of the molecular expression data (Supplementary Figures S1D, S1E).

Prior to training, we performed feature selection to retain only the most relevant information describing the separation between the control and the AD samples and to generate a robust model. First, we evaluated whether there would be a reason to perform feature selection separately on data from male and female individuals since AD presents differently in these two groups. In fact, two-thirds of AD patients are women (Snyder et al., 2016; Y; Song et al., 2020; Subramaniapillai et al., 2021). Furthermore, women have a significantly longer lifespan compared to men (Austad and Fischer, 2016). We found that the age at death of women with AD is slightly higher than that of healthy women (Figure 1B). On the contrary, the age at death of men with AD was slightly lower than that of healthy men (Figure 1C). Since our analysis indicated that there is a difference in the distribution of age at death in male and female individuals, we performed feature selection separately on the female and male sets to retain potential information on sex- and age-specific disease drivers. In order to achieve a more complete representation of the original data, we employed a combination of linear (F-statistic) and non-linear (mutual information) (Kraskov et al., 2004) statistical methods for feature selection (Supplementary Figures S2A–S2C). We then generated 27 datasets of different sizes by selecting different numbers of features (ranging between 7 and 6,360 features selected) and tested the ability of each dataset to represent the separation between the control and diseased sample groups. The separation between the sample groups (measured as the UMAP silhouette score) decreases as the number of selected features increases (Figure 1D), and the decrease markedly slows down at 1,080 selected features (Supplementary Figure S2D). This indicates that the 1,080 top features retain most of the information in the data, which separates the control and AD samples, while including additional features provides limited additional information. Consequently, we selected the 1,080 top features as input for the autoencoder model.

Overall, the framework for EnsembleOmicsAE (Figure 2A) includes four main components: 1) feature selection, which retains the proteins the abundance of which better describes the healthy or diseased brain; 2) the ensemble autoencoder model (including optimization, ensembling for model stability and reproducibility, and generation of extracted features), which takes as input the selected proteins and returns seven concise features from the model’s latent layer; and 3) the algorithm for calculating feature importance, which identifies signaling modules driving disease by iteratively perturbing the model to identify input features that are of most importance to the latent representation.

### 3.2 An ensemble of models generates stable latent representations

To identify an appropriate set of hyperparameters which would enable good model performance and avoid overfitting to the training data, we applied k-fold cross-validation (CV) to each one of five independent sets extracted from the whole dataset (Supplementary Figure S3). Specifically, the dataset was split into five sets, and each time a different one was held out for testing, while the remaining four sets were pooled (the training set). For every training set, we applied five-fold CV to test 40 different combinations of hyperparameters using the library Optuna, which efficiently navigates the hyperparameter space (Akiba et al., 2019). This approach identifies sets of hyperparameters that consistently perform well, regardless of how the data are split and are particularly adept at optimizing models which rely on a relatively small number of samples (Beebe-Wang et al., 2021; Wong, 2015). A first round of hyperparameter tuning was performed by setting up an Optuna study with the objectives of minimizing reconstruction loss and maximizing the separation of sample groups in the autoencoder latent layer (measured with the UMAP silhouette score of the embedding extracted from the autoencoder latent layer). The ranges for the hyperparameters were as follows: number of neurons in the first hidden layer (between 128 and 512); number of neurons in the second hidden layer (between 32 and 256); number of neurons in the latent layer (between 4 and 10); learning rate (between 5e-5 and 1e-3); lambda regularization L1 (between 1e-6 and 1e-3); and lambda regularization L2 (between 1e-5 and 1e-3). In total, the study was set to conduct 1,000 trials across the five data splits and the iterations of 5-fold CV within each. Up to the top-three Pareto-optimal solutions (Jin and Sendhoff, 2008) for each data split were selected, for a total of 13 solutions, and used to train on the full data set (Supplementary Table S4). When the resulting models were examined, some were found to be overfit to the training data despite the fact of having low reconstruction errors (Supplementary Figures S4A and S4B). We therefore selected new ranges of hyperparameter values to be employed for a second round of optimization based on the models that were not overfitting (Supplementary Figure S4C).

The second round of hyperparameter tuning was conducted as described above, but the ranges for the hyperparameter values were as follows: number of neurons in the first hidden layer (between 192 and 512); number of neurons in the second hidden layer (between 32 and 128); number of neurons in the latent layer (between 4 and 7); learning rate (between 1e-4 and 1e-3); lambda regularization L1 (between 2e-5 and 1e-4); and lambda regularization L2 (between 1e-4 and 1e-3). As before, we trained on the full dataset with the top solutions for each data split (18 solutions, Supplementary Table S5), and the top-performing set of hyperparameters was selected for the final model (Supplementary Table S6).

The architecture of the autoencoder model consists of 225 neurons in the first hidden layer, 128 neurons in the second hidden layer, and 7 neurons in the latent layer (Figure 2A). Individual models trained with the chosen set of hyperparameters were shown to not overfit to the training data (Figure 2B). Finally, we wanted to determine the ability of individual models compared with ensemble models to generate a stable latent representation, which is necessary for deriving a consistent biological interpretation. To this end, we trained and predicted using 100 individual models and 10 ensemble models, each derived from combining 10 individual models. We found that the sets of latent features extracted from the ensemble models are significantly more reproducible across models than those extracted from the individual models (Figure 2C; Supplementary Figure S5A). These results indicate that the ensemble models generate a stable set of latent features and are well-suited for downstream biological interpretation.

### 3.3 Features that are important for the model’s latent representation are enriched in biologically relevant networks

In neural networks, shallower layers detect simple features and feed them to deeper layers, which then detect increasingly complex features. This concept was first established in the dominion of image recognition, where pixels are used as inputs, and the neurons within increasingly deep layers detect simple lines and edges first, then object parts (eyes, ears, wheels, etc.), and then whole objects (faces, bikes, cars, etc.) (Lee et al., 2009). The input of EnsembleOmicsAE includes 1,080 protein expression features, so we hypothesized that the neurons within progressively deeper layers of the decoder learn to detect increasingly complex combinations of proteins. Specifically, shallower layers would learn to detect small networks of proteins that function together and feed them to deeper layers, which, in turn, would learn to detect more complex biological features. Accordingly, the neurons within the deepest layer of the decoder (i.e., the latent layer) (Figure 2A) should be learning to detect high-level biological features, which are relevant in reconstructing the input and that which can explain the separation between the control and AD brain proteome.

To test this hypothesis, our first goal was to identify the sets of proteins (i.e., the input features) that are important for describing the high-level biological features in the latent layer of our model (i.e., the latent features). Although existing methods rank the importance of input features only in relation to the model as a whole (Ivanovs et al., 2021), our goal was to identify proteins that are important for each neuron in the latent layer and test whether they each have a distinct biological identity. To do so, we devised and implemented a method which calculates importance scores by iteratively scrambling every individual input feature and estimating the impact on each one of the latent features (see Methods section). Proteins which impacted most of the model’s latent features were labeled as important (Supplementary Figure S5B). The sets of important proteins were found to be significantly enriched in biologically relevant protein–protein interactions. However, no enrichment was found in sets of proteins of similar size that were selected at random from the same data (Figure 3A). Given the significant enrichment in the protein–protein interaction within the sets of proteins identified using our algorithm, we reasoned that such sets of proteins have a functional significance in cellular signaling and will refer to them as autoencoder (AE) signaling modules (Figure 2A; Supplementary Table S7). Importantly, the feature importance algorithm identifies several well-studied drivers of AD within the AE signaling modules, including the protein Tau (MAPT) which is hyperphosphorylated and deposits intracellularly to form neurofibrillary tangles in AD (Naseri et al., 2019); the glial fibrillary acidic protein (GFAP), one of the primary intermediate filament proteins in astrocytes, which is highly increased in AD and is a marker for neuroinflammation and abnormal astrocyte activation in response to neuronal damage (Abdelhak et al., 2022); and several proteins that contribute to synaptic alterations, including the intermediate filament moesin (MSN), which promotes neurotoxicity mediated by Tau (Rayaprolu et al., 2020), among others (Supplementary Figure S5B).

To determine whether our method for feature importance identifies different proteins compared with those that would be found when using linear methods (i.e., correlation and distance metrics), we applied clustering analysis on the set of 1,080 proteins that were used as inputs for the autoencoder. We found a significantly lower overlap between the AE signaling modules and the clusters obtained using linear methods, compared with the overlap between the clusters obtained with different linear methods, including correlation, distance, and the network-based approach WGCNA (Langfelder and Horvath, 2008) (Figure 3B). Using Gene Ontology, we found that distinct biological processes are enriched across the AE signaling modules (Supplementary Figure S5C, D; Supplementary Figure S5D; Supplementary Table S8). Furthermore, the AE signaling modules include mostly distinct sets of proteins (Supplementary Figure S5E). Processes related to integrin signaling, cell adhesion, synaptic changes, oxidative stress, and altered splicing are over-represented in the AE signaling modules (Figure 3D) but not in the correlation-based clusters (Supplementary Figure S5F; Supplementary Tables S9, S10). Specifically, modules 1 and 3 are enriched in synaptic proteins involved with reduced synaptic plasticity, facilitating tau-induced neurotoxicity and promoting cognitive decline (Beckmann et al., 2023; Bereczki et al., 2016; Tucsek et al., 2017). Module 4 comprises proteins involved with the de-regulation of apoptosis, including the glycogen synthase kinase-3 (GSK3B), which has been shown to enhance apoptosis in areas of the brain that are crucial to memory and learning (Llorens-Martín et al., 2014). Modules 5 and 6 are broadly involved with changes in the response to oxidative stress and mitochondrial metabolism (Singh et al., 2019). Module 7 includes several proteins involved with altered splicing regulation, which has been shown to lead to the formation of disease-promoting isoforms of Tau (Qian et al., 2011) and of the myeloid cell surface antigen CD33 (van Bergeijk et al., 2019). Intriguingly, module 2 is enriched in integrin signaling, the alteration of which has so far not been extensively linked with AD, warranting further investigations. In contrast, the correlation-based clusters are enriched in processes that are mostly distinct from those highlighted by the autoencoder model, including transcriptional and mitochondrial regulation, the proteasome, the ribosome, and glutamatergic signaling (Supplementary Figure S5F; Supplementary Table S10).

Altogether, these findings demonstrate that the feature importance algorithm within DeepOmicsAE identifies biologically relevant sets of proteins that are specific to each neuron in the latent layer, demonstrating that each neuron in the latent layer has learned distinct biological features. We represent the biological features learned by the autoencoder as AE signaling modules show that they are distinct from the clusters derived from the application of linear methods and that they identify both established drivers of AD and provide novel insights for further investigations.

### 3.4 AD is characterized by divergent cellular processes compared with healthy aging

AD is classified into early onset or late onset based on an age cut-off, typically set at 65 years, and the presence of specific mutations. In fact, most of the cases of early-onset AD are due to genetic causes (mutations in the amyloid precursor protein, APP, and the presenilins 1 and 2, PSEN1 and PSEN2 genes) (Cacace et al., 2016), while a minority has been categorized as non-Mendelian (Reitz et al., 2020). On the other hand, late-onset AD is considered a sporadic disease with a heterogeneous etiology and lower heritability. Late-onset AD has been described as a single category of disease; however, a precise molecular characterization of the disease in different age groups is lacking and it is not clear whether different signaling nodes may be activated depending on age within the group of late-onset AD patients. About 99% of all AD cases are classified as late-onset AD (AD facts and figures, 2024) and the data analyzed in the present study are uniquely derived from late-onset AD cases. In our analysis, we found a difference between the age at death in female and male AD patients (Figures 1B, C), suggesting that distinct regulatory patterns may indeed be involved in the progression of AD depending on the patients’ age and sex. To determine the relation between each AE signaling module and the patients’ sex and age groups, we first visualized the modules using heatmaps based on hierarchical clustering. We found that each AE signaling module includes both up- and downregulated proteins when comparing the expression levels in the AD *versus* the control subjects (Supplementary Figure S6). Therefore, we applied hierarchical clustering to group the proteins within each AE signaling module into four clusters (Supplementary Figure S6), before examining their expression levels over the patients’ sex and age groups (Supplementary Figure S7). We found that sets of proteins in AE signaling module no. 7 are differentially regulated depending on the individuals’ sex (Supplementary Figures S8A, S8B). GO terms related to mRNA splicing are highly enriched within this set of proteins (Supplementary Figures S9A, S9B), in line with existing reports on differential regulation of splicing in female compared with male AD patients (Olesnicky et al., 2023). Regarding the association of age with signaling in AD, we found that the proteins in the AE signaling module nos 4 and 6 are differentially regulated in AD *versus* control brain samples depending on the age of the individuals they were derived from Figure 4A. In particular, clusters 1, 2, and 4 in the AE signaling module no. 4 and clusters 1 and 3 in the AE signaling module no. 6 show the most differential patterns of regulation over age when comparing the control and AD samples (Figure 4B; Supplementary Figure S10A). Recent studies have shown that plasma protein expression levels change during aging and identified protein signatures that predict health and disease in older age (Oh et al., 2023; Walker et al., 2023). Therefore, we reasoned that it would be important to discriminate between changes that are specific to the AD brain and those that are part of healthy aging. To do so, we compared the relative protein expression level changes over aging in healthy and AD subjects and identified a subset of proteins within the AE signaling module nos 4 and 6, for which the changes over age are primarily occurring in AD but not in healthy individuals (AD-dominant effect) (Figure 4C; Supplementary Figure S10B). This analysis highlighted a larger set of proteins regulated over age with an AD-dominant effect in the AE signaling module no. 4, and we therefore focused on this module for the subsequent analyses. We found that the Bergmann glial cell differentiation process is significantly enriched within the proteins regulated over age with an AD-dominant effect in the AE signaling module no. 4 (Supplementary Figure S10C). Conversely, multiple metabolic functions including the pyruvate metabolic process and the pentose phosphate shunt are enriched within the set of proteins that are regulated more prominently during healthy aging (aging-dominant effect) (Supplementary Figure S10D).

Among the proteins in the AE signaling module no. 4 that are regulated in an AD-dominant manner and involved in a cellular differentiation process, we identify vimentin (VIM) and the mitogen-activated protein kinases 1 and 3 (MAPK1 and MAPK3) (Figure 4C). VIM is a component of the intermediate filaments and a marker of the reactive astrocytes which surround amyloid plaques in AD (Hol and Pekny, 2015). VIM and MAPK1 and MAPK3 act coordinately in adult neurons to mediate neurite extension during repair of nerve damage (Levin et al., 2009; Perlson et al., 2005). VIM is also expressed by differentiating neurons in the human fetal brain (Ho and Liem, 1996), where it cooperates with the MAPKs to facilitate neurite extension and establish new synaptic connections (Boyne et al., 1996). VIM levels slightly increase during healthy aging, however, not significantly (Figure 4D). In AD, we found that VIM expression levels are elevated compared with healthy samples (Figure 4E) and that the difference is greater in younger compared with older individuals both at the protein level (Figure 4F) and at the transcript level in a larger cohort of samples (Supplementary Figure S11A). The same trend is observed for MAPK1 and MAPK3 (Supplementary Figures S11B and S11C). Finally, we found that the pathological staging and cognitive function is similar between the AD patients that died at a younger age and those that died at an older age (Supplementary Figure S11D), indicating that the two subsets are pathologically undistinguishable.

## 4 Discussion

The analysis and interpretation of large sets of omics data offers the opportunity to gain a holistic understanding of molecular regulation dynamics and their contribution to disease progression. Autoencoders are a powerful tool for reducing the dimensionality of omics data, thereby facilitating the extraction of key biological information. They comprise multiple layers of neurons, each containing a non-linear activation function, which allows them to capture non-linear, hierarchical, and multi-modal relationships within the data (Bank et al., 2023). However, the biological interpretation of complex models based on neural networks such as the Autoencoders has proved to be challenging (Ching et al., 2018). Broadly, approaches for determining feature importance can be classified as perturbation-based or backpropagation-based. Such methods include permutation feature importance (PFI) (Altmann et al., 2010), SHapley Additive exPlanations (SHAP) (Antwarg et al., 2019), and integrated gradients (IGs) (Sundararajan et al., 2017), all of which rank feature importance relative to the entire model. Here, we introduce a perturbation-based approach that outputs feature importance relative to individual neurons in the autoencoder latent layer. Previously, efforts in this direction include the development of an approach to identify the components of an image that each neuron is detecting, based on the generation of visual inputs that would maximally activate specific neurons in the network (Bengio et al., 2009; Erhan et al., 2009). In the biomedical field, this method was applied to identify transcription factor-binding motifs when analyzing genomic sequencing data (Finnegan and Song, 2017). Here, we sought to interpret the contributions of individual neurons in order to characterize whether they detect distinct signaling units driving disease. We demonstrate that our approach identifies biologically relevant sets of proteins (Figure 3A) that are not found using linear methods such as the correlation or distance metrics (Figure 3B). Several known AD drivers were identified within the AE signaling modules, including the protein Tau (MAPT), GFAP, MSN, and other proteins involved with synaptic alterations (Figure 3D; Supplementary Figure S5B). Other proteins that are well-studied in the context of AD, however, were not identified, including the apolipoprotein E (APOE) and the amyloid precursor protein (APP). In humans, there are three common alleles for the gene encoding the apolipoprotein E (APOE2, APOE3, and APOE4). These alleles give rise to six possible genotypes, namely, APOE2/2, 2/3, 2/4, 3/3, 3/4, and 4/4, of which APOE3/3 is the most common (about 62% of the population) (de-Almada et al., 2012) and is known to not affect the risk for AD. Instead, carrying one or two APOE2 alleles protects from developing AD (Reiman et al., 2020), whereas APOE4 increases the risk to develop AD, with each additional APOE4 copy increasing the risk and reducing the age at onset of the disease (APOE4 homozygotes, which are less than 2% of the population, have a 60% chance of developing AD by the age of 85) (Fortea et al., 2024; Genin et al., 2011). In other words, the risk to develop AD is modified by the allelic makeup of the APOE gene rather than based on its protein expression levels. Additionally, the cohort examined in the study reflects the general population heterogeneity for APOE, with 59% of the individuals being APOE3 homozygotes, 26% carrying one APOE4 allele, and only 1.7% APOE4 homozygotes (Supplementary Table S11). APOE was not necessarily expected to be identified by the model as one of the drivers since EnsembleOmicsAE is trained on protein abundances and not genetic information. The amyloid precursor protein (APP), which is proteolytically cleaved to form beta-amyloid that accumulates in AD brains, was also not identified as one of the drivers within the signaling modules. One possibility is that while EnsembleOmicsAE is adept at identifying signaling modules of proteins that are functionally associated (Figure 3A), APP is not as strongly functionally associated with other proteins in AD, and for this reason, it may not have been selected. Specifically, functional association includes either proteins that contribute to the same biological process or pathway, are associated via transcriptional regulation (i.e., a transcription factor and its targets), are co-expressed, or are part of the same protein complex. However, the pathogenic role of beta-amyloid accumulation has been linked to a plethora of factors that are not necessarily linked with the functional roles of APP itself, such as brain inflammation, defective clearance, and alterations in the blood–brain barrier (Hampel et al., 2021). Furthermore, transcriptional or translational de-regulation of APP expression levels is not a universal feature of AD. Although AD patients who carry the APOE4 allele have an increased expression of APP due to transcriptional upregulation (Huang et al., 2017), most AD patients in the examined cohort do not carry APOE4 (>60%). Additionally, while approximately 16% of the healthy individuals did carry the APOE4 allele, leading to transcriptional upregulation of APP, they did not develop the disease. Therefore, rather than APP directly participating in regulatory networks, multiple factors affect the extent to which beta-amyloid is toxic in the brain, and additionally, the regulation of APP transcription is not strongly associated with disease status. Collectively, there are multiple opportunities for improving on the framework presented in this study. One is that of expanding its applicability to include genetic data and molecular expression data, to more comprehensively capture key drivers of disease. Additionally, while we have previously presented a first attempt to integrate clinical information into the model (Panizza, 2023), we believe that improvements will be necessary for the feature importance algorithm to better perform with the wide variety of value distributions that are characteristics of clinical features. Finally, we show that EnsembleOmicsAE contributes novel information as the AE signaling modules are distinct from the clusters based on linear methods (Figure 3D; Supplementary Figure S5E) and are enriched in cellular processes that have not previously been linked with AD, namely, cell adhesion mediated by integrins and the integrin complex (Figure 3D), thus expanding the current narrative on the molecular determinants of AD progression.

In our study, we then related the AE signaling modules with the age at death of the patients (Supplementary Figure S7). When looking at changes in protein expression levels over age in AD compared with healthy aging, we found an enrichment of genes involved with the differentiation process (Figure 4C). In particular, younger AD patients have a greater increase in the levels of VIM compared with older AD patients (Figure 4E; Supplementary Figure S11A). The same trend is observed for MAPK1 and MAPK3, which are also part of the same signaling network involved with cellular differentiation (Supplementary Figures S11B, S11C). VIM, MAPK1, and MAPK3 work in concert to mediate neurite extension during nerve repair and during early development (Pebworth et al., 2021), and VIM is a marker of neural precursor cells (Levin et al., 2009). Levin et al., performed immunohistochemical staining to show that VIM is expressed in AD brains but not in the same regions of healthy brains. In their analysis, VIM is mainly present in dendrites and its expression level correlated with the extent of local pathology. VIM is also a mesenchymal marker, and it has been shown to cooperate with MAPK1 to regulate epithelial-to-mesenchymal transition (EMT) (Xu et al., 2018). We indeed find that multiple mesenchymal markers (Beckmann et al., 2023; Lamouille et al., 2014) are upregulated in the AD compared with the control samples, while the expression of epithelial markers was undetectable (Supplementary Figure S11E). Additionally, the expression of the transcription factor SOX2, which is a key marker for neuronal progenitor, and especially radial glia cells together with VIM (Pebworth et al., 2021), is also upregulated in the same set of younger AD patients (Supplementary Figure S11F), further substantiating the signature as marking a progenitor-like state. Accordingly, previous reports implicate MAPK1/3 and SOX2 in mediating EMT (Wang K et al., 2018), as well as neuronal loss and cognitive impairment in mouse models of AD (Kim et al., 2023). Altogether, our findings suggest that a potential reactivation of a developmental program involving VIM, MAPKs, SOX2, and EMT may play a role in driving AD in younger patients. Our findings open multiple questions including whether this program may lead to the de-differentiation of neurons into precursor cells, whether it might contribute to AD onset and progression, and how aging may influence the activation of this program. Further studies will be needed to determine the exact role of these events in the pathogenesis of AD. The role of VIM in triggering astrocyte over-reactivity in AD also needs to be further elucidated (Kamphuis et al., 2015). Our hypothesis that the activation of the VIM-SOX2 axis might have a differential effect on AD progression depending on the individuals’ age carries similarities with the way other pathways have been described to be differentially activated over age. One example is the expression of the genes encoding for the triggering receptor expressed on myeloid cells (TREM) that play important roles in both the innate and adaptive immunity and for which specific polymorphisms have been shown to modify the risk to develop AD. Chan et al., reported that a polymorphism of TREM1 leads to increased AD pathology and cognitive decline by raising the cell surface expression levels of the anti-inflammatory TREM2 in younger but not in older individuals (Chan et al., 2015). Another example is the finding by Lo et al. that a cluster of genes located on chromosome 19, which includes the APOE gene, has a greater ability to promote AD in younger compared with older individuals (Lo et al., 2019). Altogether, while important questions remain to be addressed, the idea that the activation of specific disease pathways may depend on the individuals’ age (Guerreiro and Bras, 2015) carries the potential in discovering novel drivers of disease and developing tailored therapeutic approaches.

In our study, we resolved the complex molecular inter-relationships learned by EnsembleOmicsAE over the age of the patients, to refine and expand our understanding of the AD pathogenesis. The combination of these two approaches (i.e., machine learning and age-differential analysis) led to identifying signaling nodes that are over-activated in younger compared with older individuals, in the context of both healthy aging and AD (Figure 4C). Our combined approach provides a framework for the age-differential analysis of large-scale data, which will be applicable as new data become available. Such time-resolved analyses will be of value not only to further characterize the differences between younger and older AD patients but also to resolve earlier *versus* later signaling events, leading to the onset and progression of AD. This is of particular relevance since AD initiates in the brain several years or even decades before clinical symptoms appear (the so-called preclinical or cellular stage) (Counts et al., 2017; De Strooper and Karran, 2016; Petersen, 2018; Preische et al., 2019; Sperling et al., 2012). Therefore, the understanding of the first steps leading to the pathology would be of great importance for preventing AD, an approach that is gaining increasing attention (Frisoni et al., 2023; Niotis et al., 2022).

In sum, the current work contributes a novel algorithm for scoring feature importance that discriminates which features are more relevant to each neuron within the latent layer of the autoencoder. It further provides an expanded understanding of molecular drivers of AD which are missed by linear methods. Finally, our work highlights age-specific relationships between the regulation of a differentiation program and AD progression and contributes to our understanding of AD pathogenesis at different ages.

## Data Availability

The brain proteomics and RNAseq datasets for this study can be found in the AD Knowledge Portal (https://adknowledgeportal.org/) using the data identifiers specified in the Materials. The plasma proteomics data is available through Oh et al. (Oh et al., 2023).
