# Supplementary material for: An interpretable deep learning framework identifies proteomic drivers of Alzheimer’s disease
Source: Front Cell Dev Biol. 2024 Sep 17;12:1379984. doi: 10.3389/fcell.2024.1379984 (PMC11442384; doi:10.3389/fcell.2024.1379984)
Supplement: Supplementary file 1 [file Presentation1.PDF]

A

Data Set	No. of proteins (total)	No. of proteins (shared)	Outcomes measured	Clinical features
Banner	7,164	6,360	Diagnosis; CERAD score; Braak score	Sex; age at death
ROSMAP Round 1	7,164	6,360		Sex; age at death; race; spanish; education
MSBB	9,209	6,360		Sex; age at death; race; ethnicity

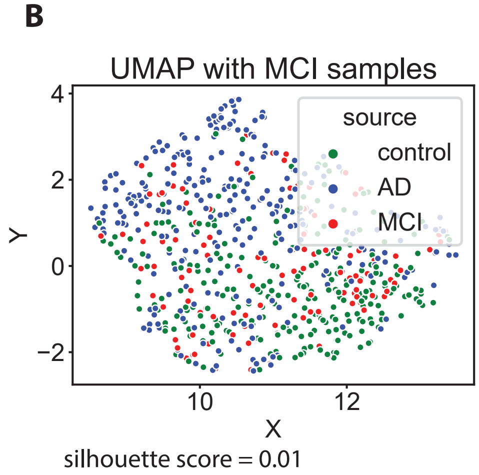

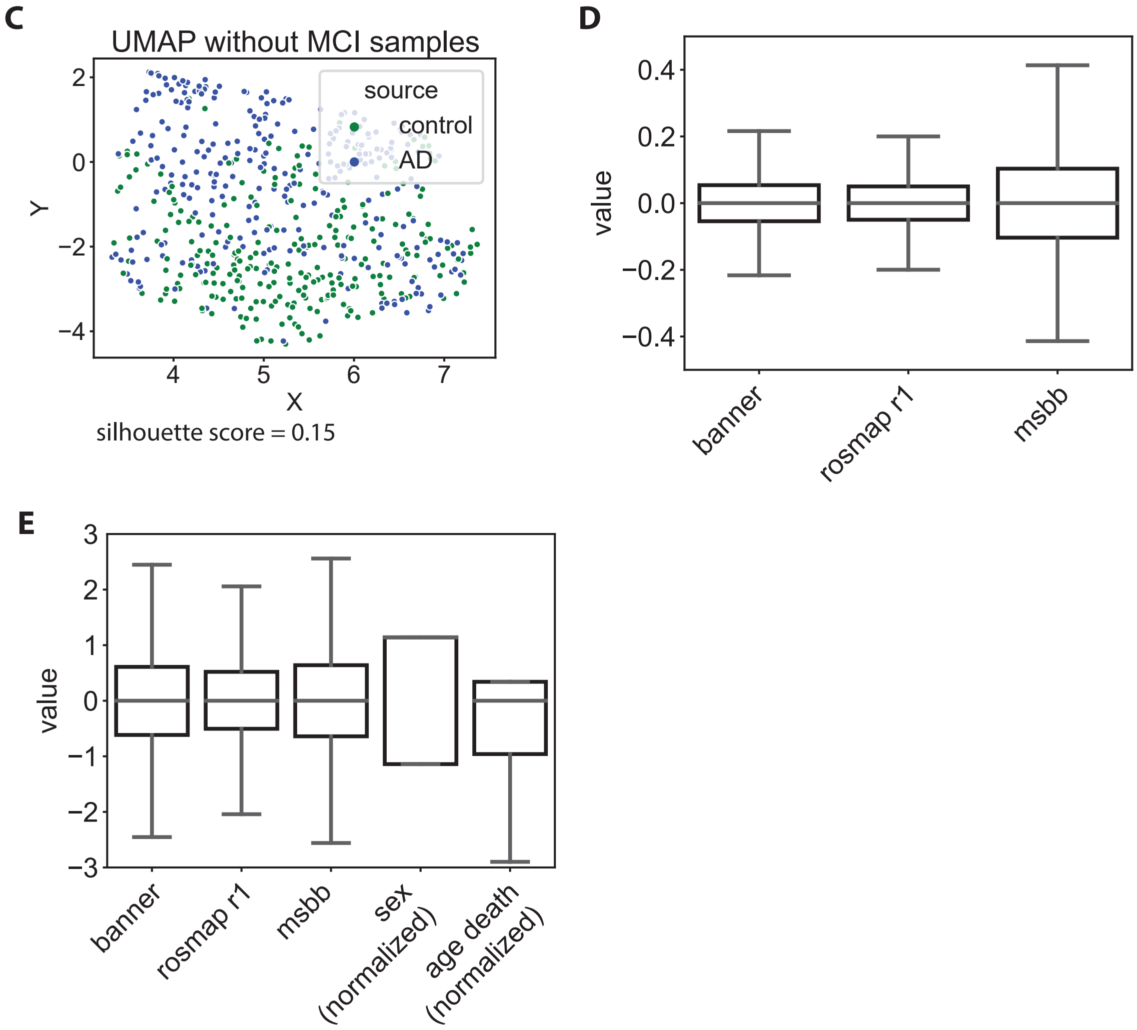

Supplementary Figure S1.

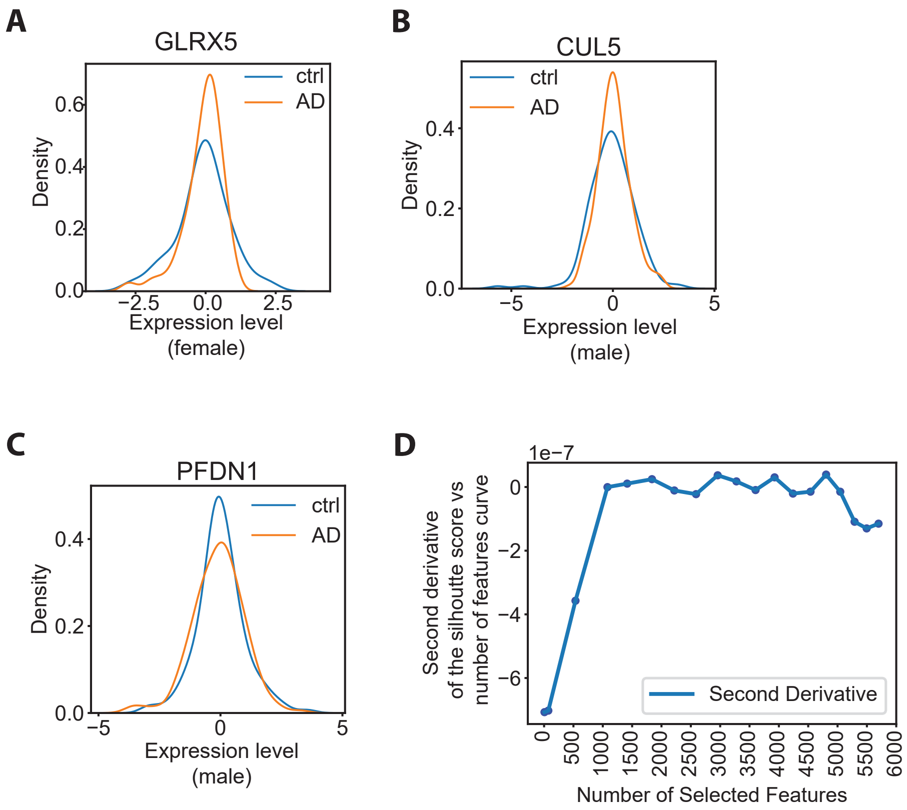

Supplementary Figure S2

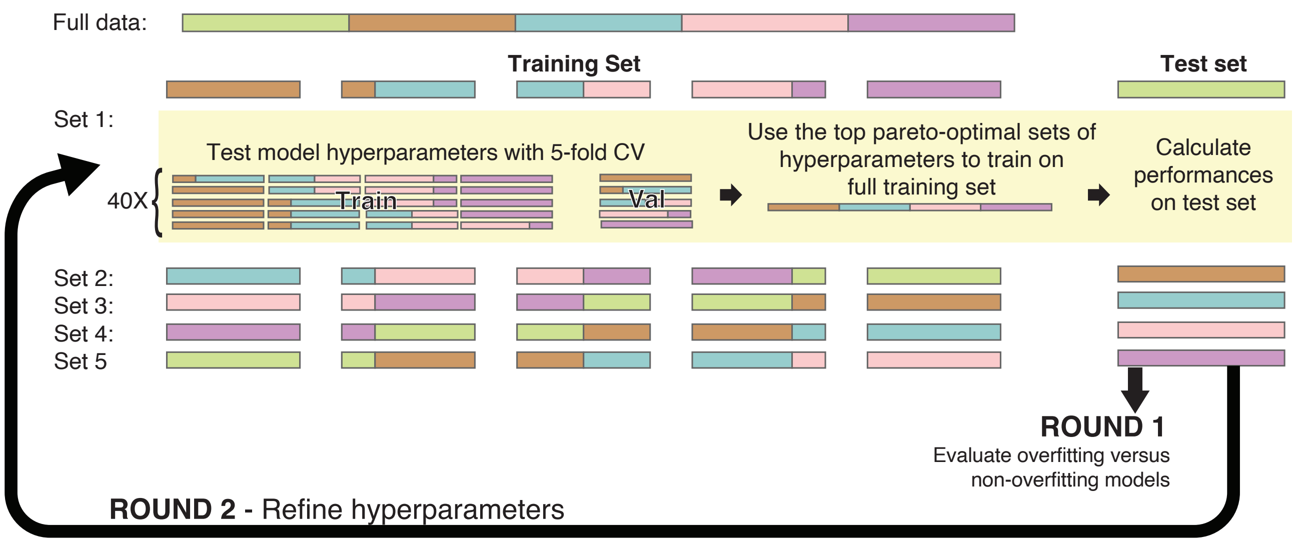

Supplementary Figure S3.

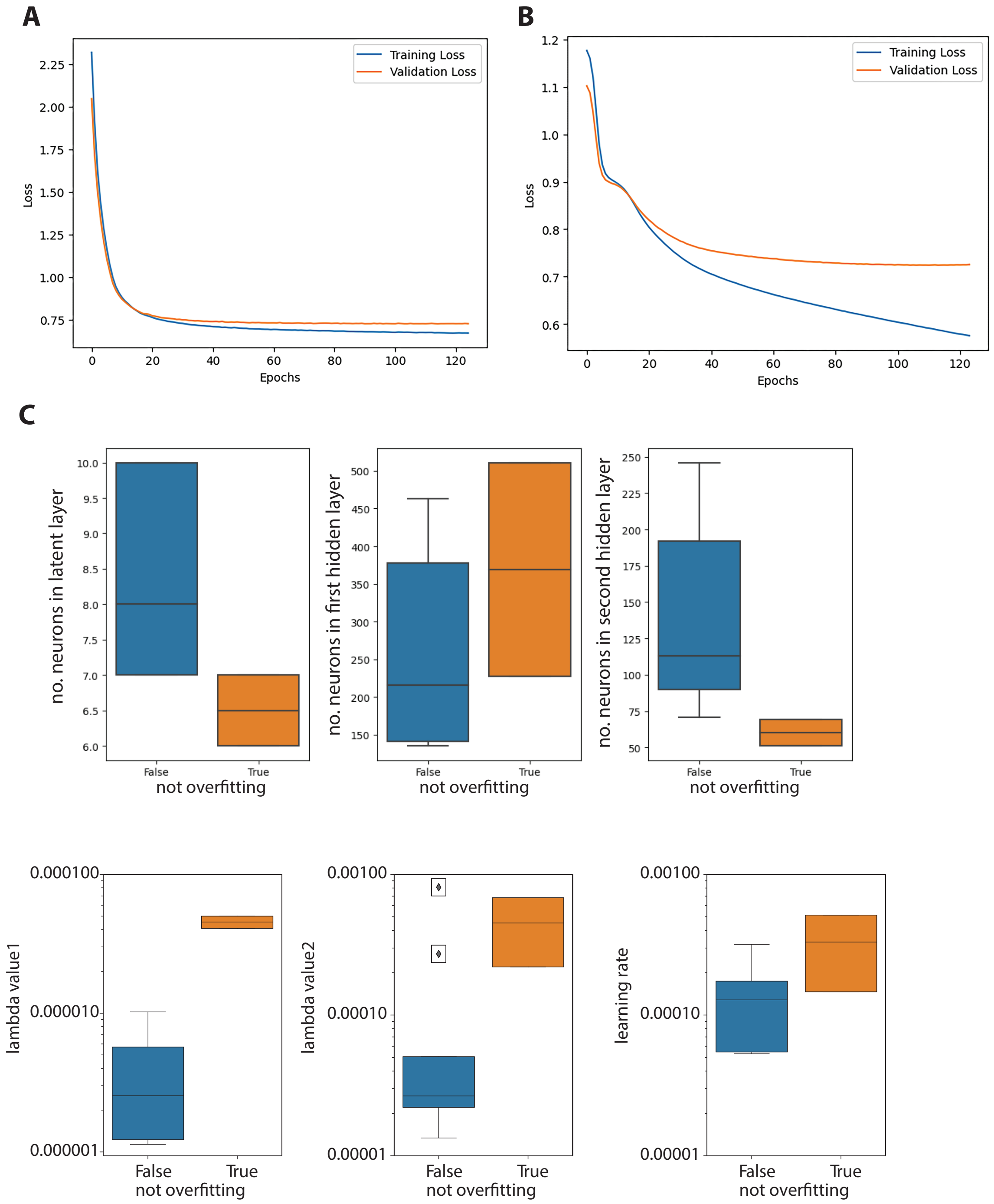

Supplementary Figure S4.

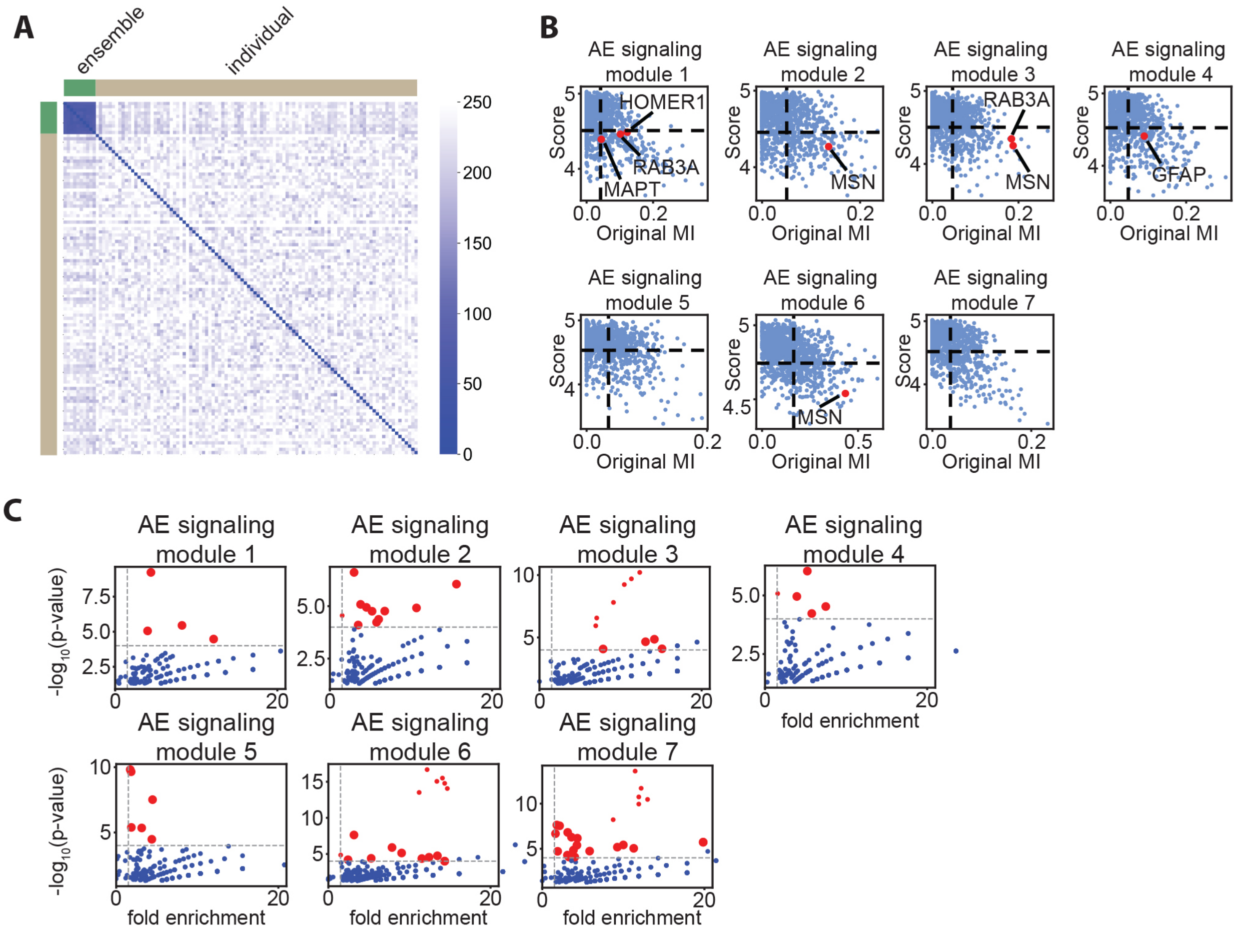

D

1.
- monoatomic cation transmembrane transport
- postsynaptic density/dendritic spine

2.
- integrin complex/cell-cell and cell-matrix adhesion
- actin cytoskeleton organization

3.
- cellular potassium ion homeostasis
- extrinsic apoptotic signaling pathway in absence of ligand

4.
- synaptic vesicle membrane
- apical plasma membrane

5.
- nucleus
- mRNA splicing, via spliceosome

6.
- electron transfer activity/mitochondrial inner membrane/mitoch. respiratory chain
- S100 protein binding

7.
- negative regulation of mRNA splicing, via spliceosome/ribonucleoprotein complex/RNA binding
- mitochondrial inner membrane/mitoch. respiratory chain

E

	module_no_1	module_no_2	identity_percent
19	4	6	38.636364
6	1	2	28.915663
7	1	3	22.891566
20	5	6	17.777778
17	3	6	14.893617
18	4	5	14.772727
9	1	5	13.253012
3	0	4	12.903226
5	0	6	12.903226
10	1	6	12.048193
1	0	2	11.827957
16	3	5	11.702128
0	0	1	10.752688
13	2	5	10.256410
4	0	5	9.677419
11	2	3	7.692308
14	2	6	7.692308
8	1	4	6.024096
15	3	4	5.319149
12	2	4	5.128205
2	0	3	3.225806

Supplementary Figure S5 (page1)

F

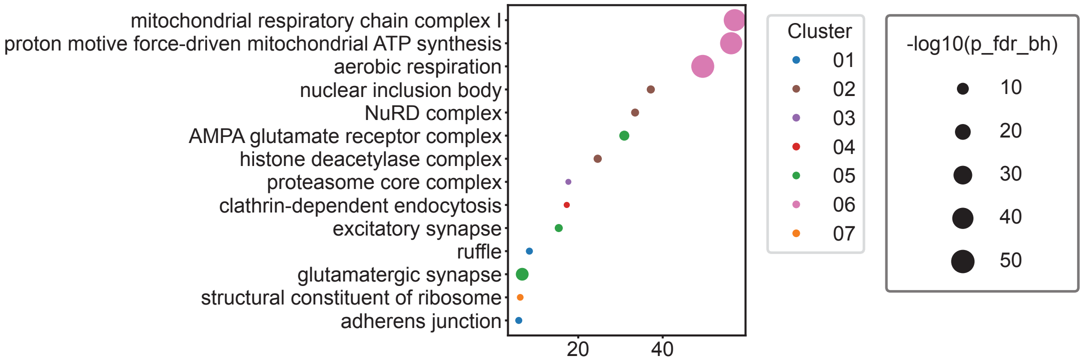

Supplementary Figure S5 (page 2)

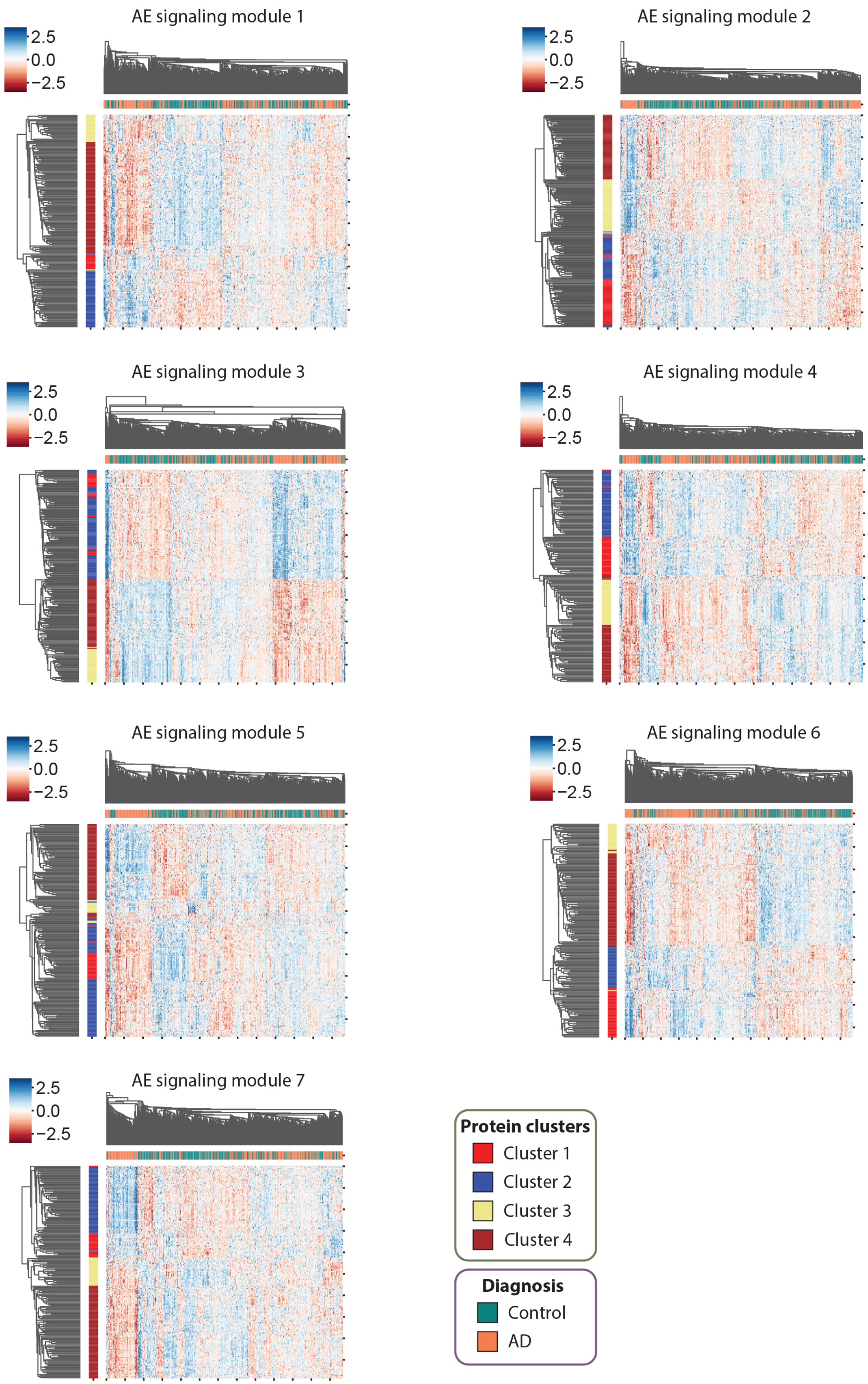

Supplementary Figure S6.

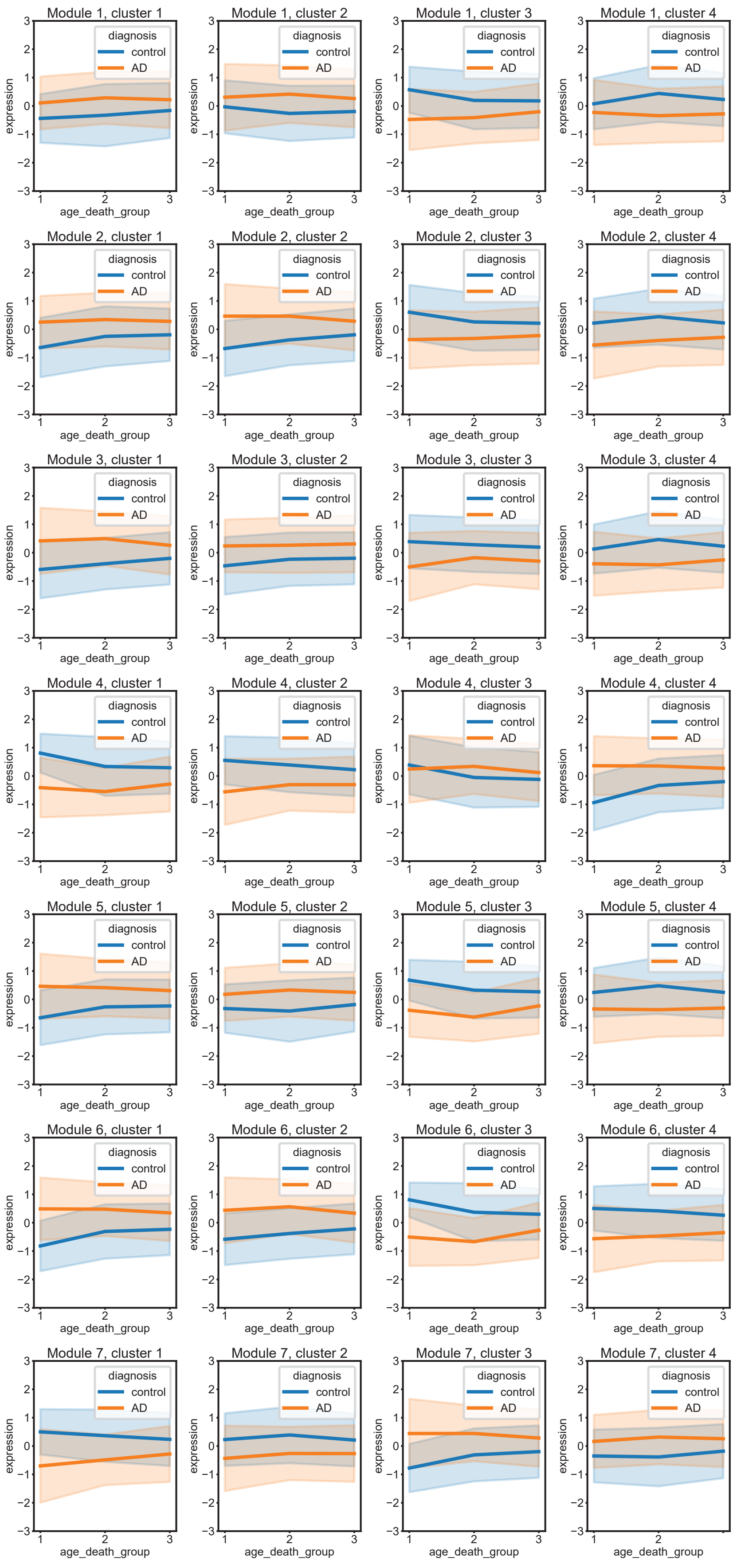

Supplementary Figure S7.

A

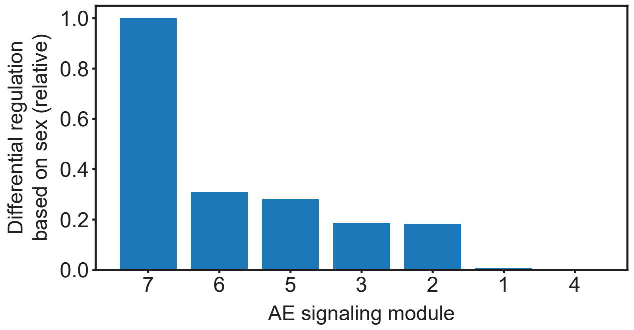

B

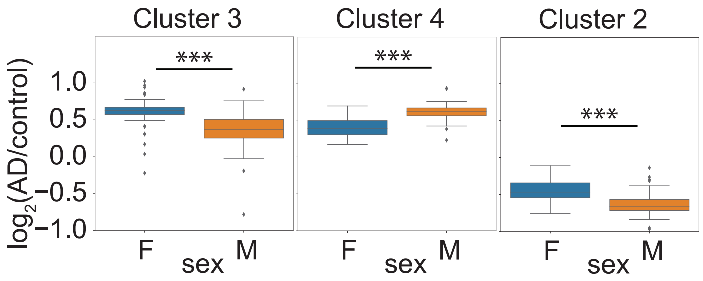

Supplementary Figure S8.

A

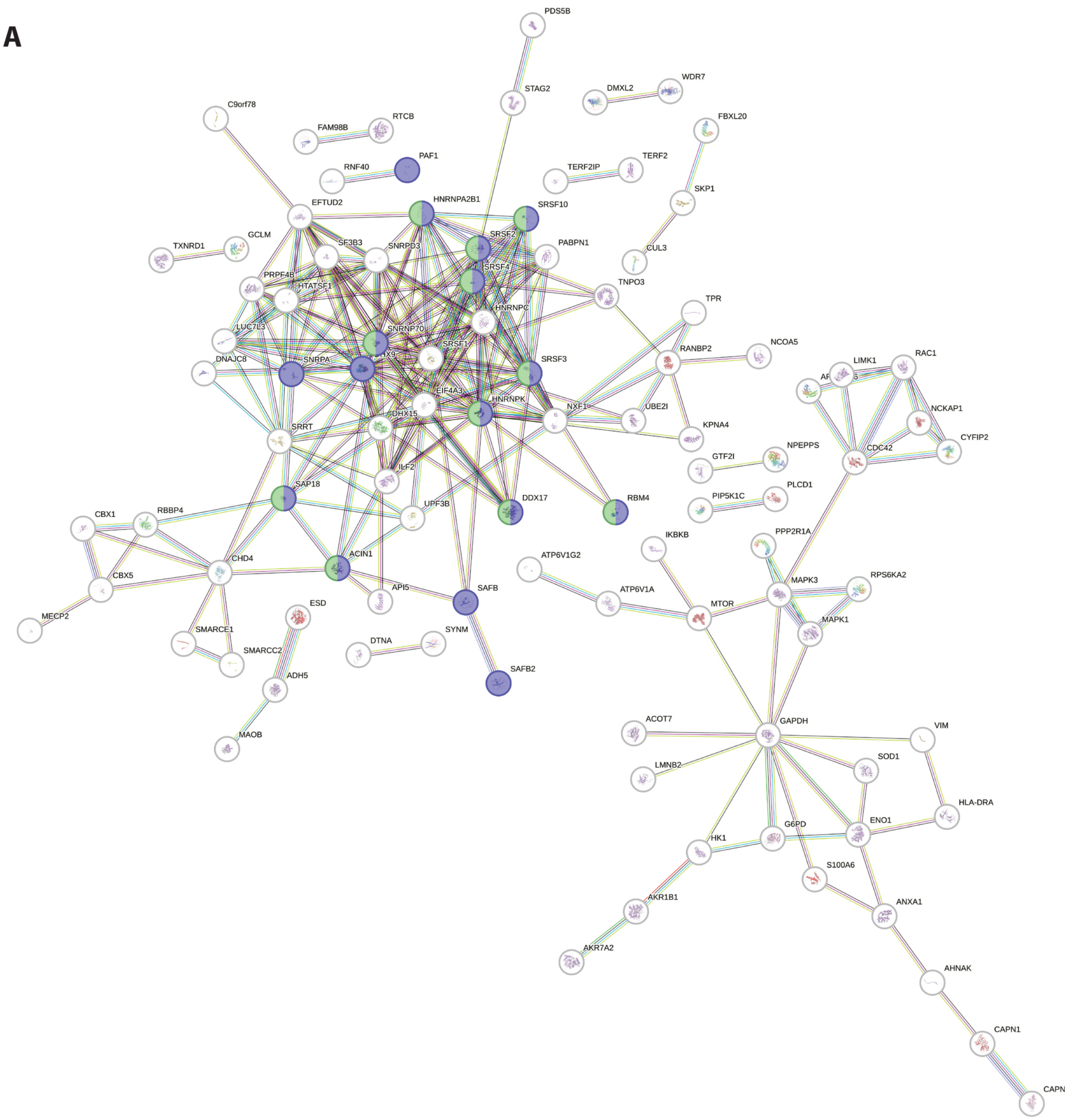

B

explain columns

Biological Process (Gene Ontology)

GO-term	description	count in network	strength	false discovery rate
GO:0048239	Negative regulation of DNA recombination at telomere	2 of 2	2.1	0.0494
GO:0060020	Bergmann glial cell differentiation	3 of 11	1.53	0.0276
GO:0044346	Fibroblast apoptotic process	3 of 11	1.53	0.0276
GO:0048025	Negative regulation of mRNA splicing, via spliceosome	6 of 24	1.5	5.62e-05
GO:0061308	Cardiac neural crest cell development involved in heart deve...	3 of 12	1.5	0.0320
GO:0031053	Primary miRNA processing	3 of 13	1.46	0.0362
GO:0010592	Positive regulation of lamellipodium assembly	4 of 29	1.24	0.0229
GO:0050684	Regulation of mRNA processing	17 of 140	1.18	1.63e-11
GO:0048024	Regulation of mRNA splicing, via spliceosome	11 of 110	1.1	2.19e-06
GO:0006406	mRNA export from nucleus	6 of 65	1.06	0.0049
GO:0000398	mRNA splicing, via spliceosome	22 of 245	1.05	1.14e-12
GO:1903312	Negative regulation of mRNA metabolic process	8 of 92	1.04	0.00039
GO:0000245	Spliceosomal complex assembly	6 of 76	1.0	0.0103
GO:0008380	RNA splicing	29 of 370	0.99	1.16e-15
GO:0051028	mRNA transport	10 of 129	0.99	7.57e-05
GO:1904356	Regulation of telomere maintenance via telomere lengthening	5 of 64	0.99	0.0330
GO:0043484	Regulation of RNA splicing	14 of 183	0.98	4.45e-07
GO:0032206	Positive regulation of telomere maintenance	5 of 67	0.97	0.0374
GO:1903311	Regulation of mRNA metabolic process	21 of 302	0.94	2.16e-10
GO:0051168	Nuclear export	9 of 132	0.93	0.00054
GO:0006913	Nucleocytoplasmic transport	16 of 248	0.91	3.29e-07

Supplementary Figure S9.

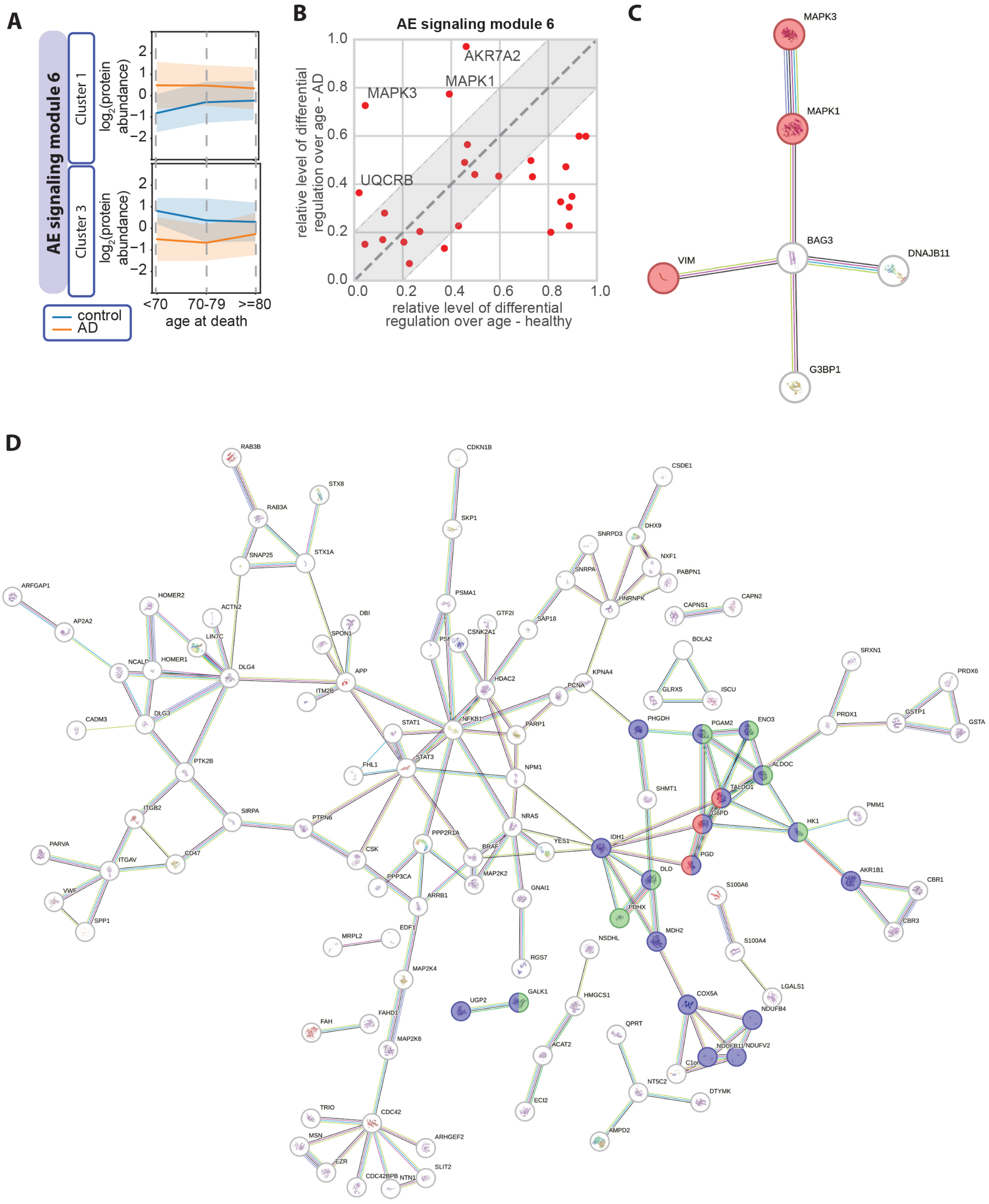

Supplementary Figure S10.

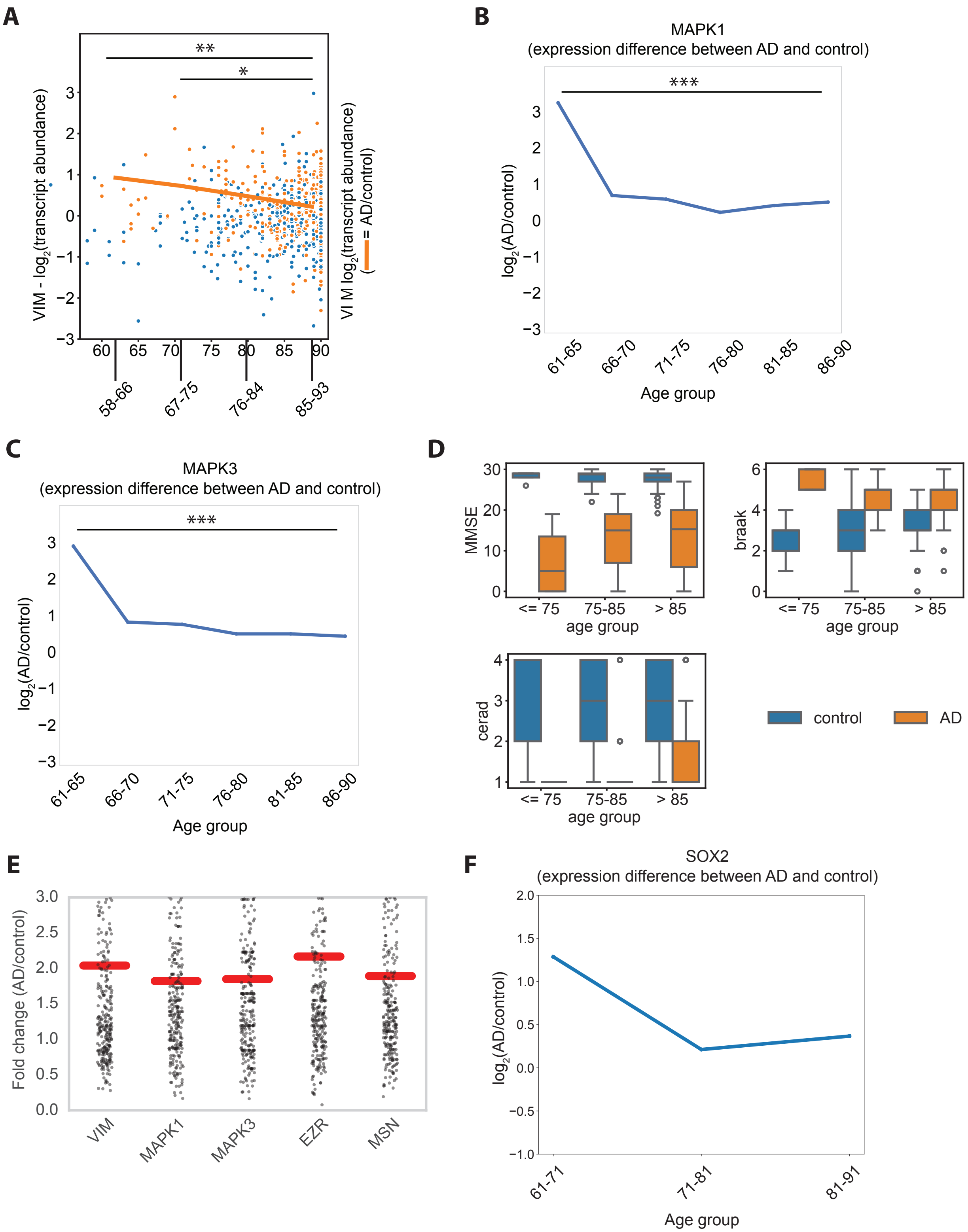

Supplementary Figure S11.
